# Spatial transcriptomics reveal neuron–astrocyte synergy in long-term memory

**DOI:** 10.1038/s41586-023-07011-6

**Published:** 2024-02-07

**Authors:** Wenfei Sun, Zhihui Liu, Xian Jiang, Michelle B. Chen, Hua Dong, Jonathan Liu, Thomas C. Südhof, Stephen R. Quake

**Affiliations:** 1 Department of Bioengineering, Stanford University, Stanford, CA, USA.; 2 Department of Molecular and Cellular Physiology, Stanford University School of Medicine, Stanford, CA, USA.; 3 Howard Hughes Medical Institute, Stanford University School of Medicine, Stanford, CA, USA.; 4 Institute for Stem Cell Biology and Regenerative Medicine, Stanford University School of Medicine, Stanford, CA, USA.; 5 Chan Zuckerberg Initiative, Redwood City, CA, USA.; 6 These authors contributed equally: Wenfei Sun, Zhihui Liu.

## Abstract

Memory encodes past experiences, thereby enabling future plans. The basolateral amygdala is a centre of salience networks that underlie emotional experiences and thus has a key role in long-term fear memory formation^1^. Here we used spatial and single-cell transcriptomics to illuminate the cellular and molecular architecture of the role of the basolateral amygdala in long-term memory. We identified transcriptional signatures in subpopulations of neurons and astrocytes that were memory-specific and persisted for weeks. These transcriptional signatures implicate neuropeptide and BDNF signalling, MAPK and CREB activation, ubiquitination pathways, and synaptic connectivity as key components of long-term memory. Notably, upon long-term memory formation, a neuronal subpopulation defined by increased *Penk* and decreased *Tac* expression constituted the most prominent component of the memory engram of the basolateral amygdala. These transcriptional changes were observed both with single-cell RNA sequencing and with single-molecule spatial transcriptomics in intact slices, thereby providing a rich spatial map of a memory engram. The spatial data enabled us to determine that this neuronal subpopulation interacts with adjacent astrocytes, and functional experiments show that neurons require interactions with astrocytes to encode long-term memory.

Consolidation of newly acquired memories into long-term memories and reconsolidation of long-term memory during recall requires transcription and translation, as shown by extensive studies of the role of gene expression during learning and memory^2,3^. Although key transcription factors in learning and short-term memory, such as CREB^4^, have been identified, the overall nature of long-term memories, which can persist for a lifetime, remains unknown. Gene-expression changes are known to be essential for long-term memory, but the cell types and the nature of the transcriptional programmes involved are incompletely understood. Moreover, multiple brain regions have been implicated in long-term memory formation and storage but it is unknown whether similar transcriptional processes are used in different regions of the brain.

Here, we performed high-resolution spatial and single-cell transcriptomics to comprehensively analyse the changes in the transcriptomic landscape during long-term memory formation in mice. We identified memory-specific gene-expression changes in the amygdala, a complex brain region within which the basolateral amygdala (BLA) is implicated in short- and long-term memories associated with salient experiences, such as fear. Lesions of the BLA abolish both short-term and long-term fear memory^5^. In fear learning paradigms, suppressing RNA transcription in the BLA before training attenuates fear memory consolidation without affecting the freezing response to a foot shock^6^. Inhibiting protein synthesis in the BLA immediately after training^7^ or after reactivation^8^ also impairs long-term memory consolidation, but does not affect short-term memory recall^8^.

Our results show that neurons and astrocytes in the BLA exhibit memory-specific persistent transcriptional signatures that correspond to multiple signalling pathways but are highly specific to a small subset of cells that represent engram cells. We identified a subpopulation of neurons with increased *Penk* and decreased *Tac* expression (P^+^T^−^ neurons) that constitute the most prominent part of the long-term memory engram. Using spatial transcriptomics, we discovered a population of astrocytes that are adjacent to P^+^T^−^ neurons, undergo gene-expression changes in forming long-term memory, and are required for long-term memory consolidation. Finally, integration of these results with previous data^9^ on long-term contextual fear memory in the medial prefrontal cortex enabled us to examine region-specific versus general gene expression changes. This integration revealed that similar molecular programmes and cell types are used in long-term fear memories across both regions of the brain.

## Persistent changes during fear memory recall

**Fig. 1 |:**
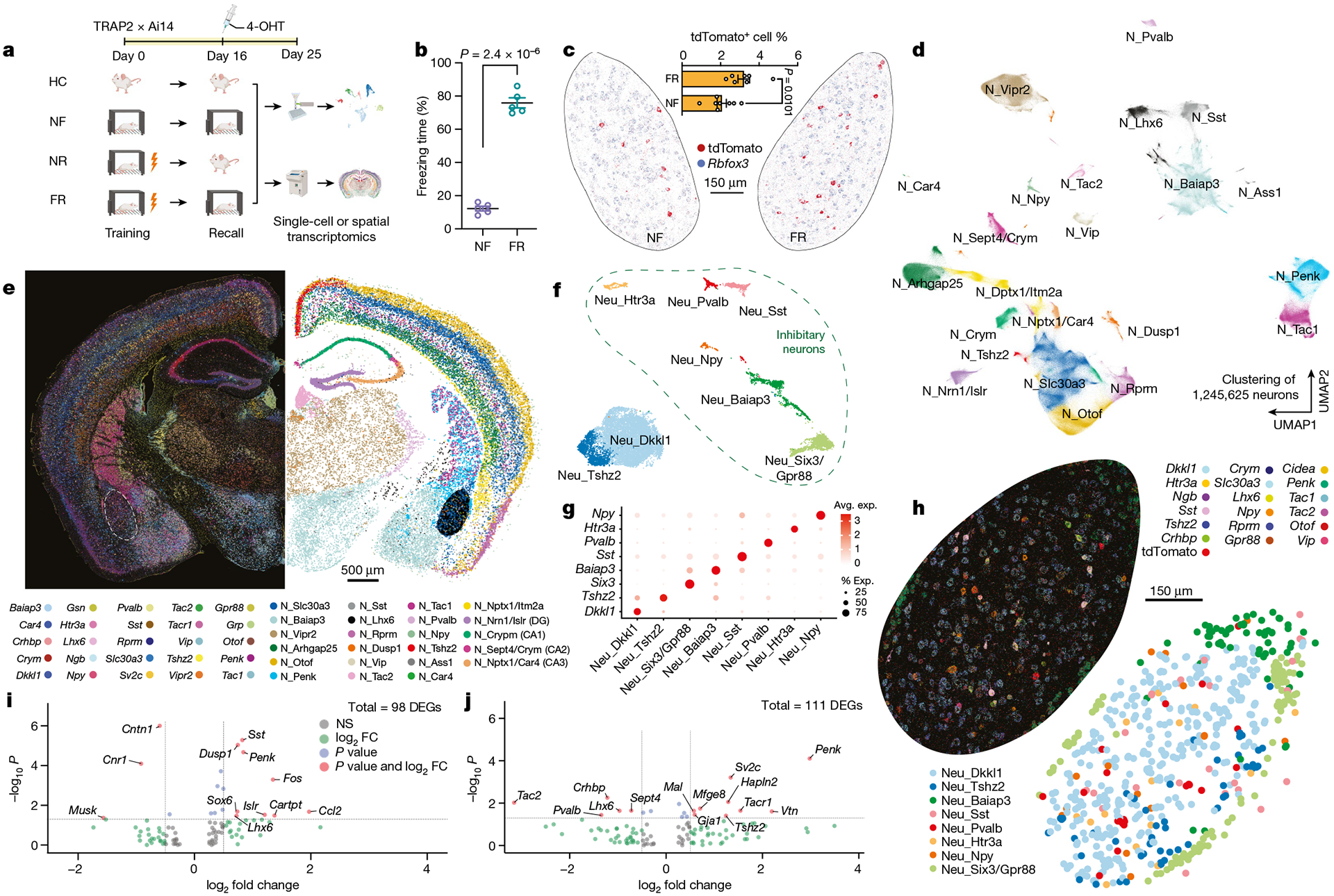
Spatial transcriptomics resolves the engram assembly and memory-associated genes. **a**, Experimental scheme for tracing engram cells in a fear conditioning model. Active cells during the return phase were permanently tagged with tdTomato and used for differential analyses of engram cells. 4-OHT, 4-hydroxytamoxifen. **b**, Freezing rate during the recall phase. *n* = 5 mice; data are mean ± s.e.m.; unpaired two-tailed Student’s *t*-test, *P* = 2.4 × 10^−6^. **c**–**j**, Multiplexed error-robust fluorescence in situ hybridization (MERFISH) data. **c**, Engram cells (tdTomato^+^) in BLA revealed by MERFISH. FR: *n* = 8 sections, NF: *n* = 7 sections; data are mean ± s.e.m.; unpaired two-tailed Student’s *t*-test. **d**, Unbiased clustering of all neurons. **e**, Neuronal markers and cell-type annotations resolved in space. **f**, Unbiased clustering of neurons within BLA. **g**, Marker genes of BLA neuronal subtypes. Avg., average; exp., expression. **h**, Neuronal markers and cell-type annotations of BLA. **i**, Fear memory-induced gene expression in excitatory engram neurons of BLA. *P* < 0.05, unadjusted *P* value by Mann–Whitney–Wilcoxon test. FC, fold change; NS, not significant. **j**, Fear memory-induced gene expression in inhibitory engram neurons of BLA. *P* < 0.05, unadjusted *P* value by Mann–Whitney–Wilcoxon test.

In TRAP2 mice, cellular activation induces expression of tamoxifen-dependent Cre-ERT2 recombinase embedded in the *Fos* gene. As a result, TRAP2 mice crossed to Ai14 tdTomato (tdT) reporter mice express tdT only if they are both stimulated and exposed to tamoxifen, although a stochastic background activation always remains^10^. We trained TRAP2 mice crossed to Ai14 mice by fear conditioning on day 0 and triggered recall of long-term fear memories by returning the mice to the training context 16 days later with simultaneous injection of tamoxifen. We then analysed the mouse amygdala on day 25, nine days after recall, by spatial transcriptomics and full-length deep single-cell RNA sequencing (scRNA-seq) (Fig. 1). As controls for this ‘fear training and recall’ (FR) condition, we used ‘home cage’ (HC) mice that were left in their home cage, and ‘no fear’ (NF) and ‘no recall’ (NR) mice that were exposed to all manipulations except that they received either no electrical shock during training (NF) or were not subjected to the recall condition (NR). The goal of this experimental design was to mark engram cells that are activated during the recall and become tdT^+^, enabling us to identify fear-specific memory genes that are differentially expressed in these engram cells and are not induced by salience only^9^ (the NF condition).

## A spatially resolved ensemble of engram cells

**Extended Data Fig. 1 |:**
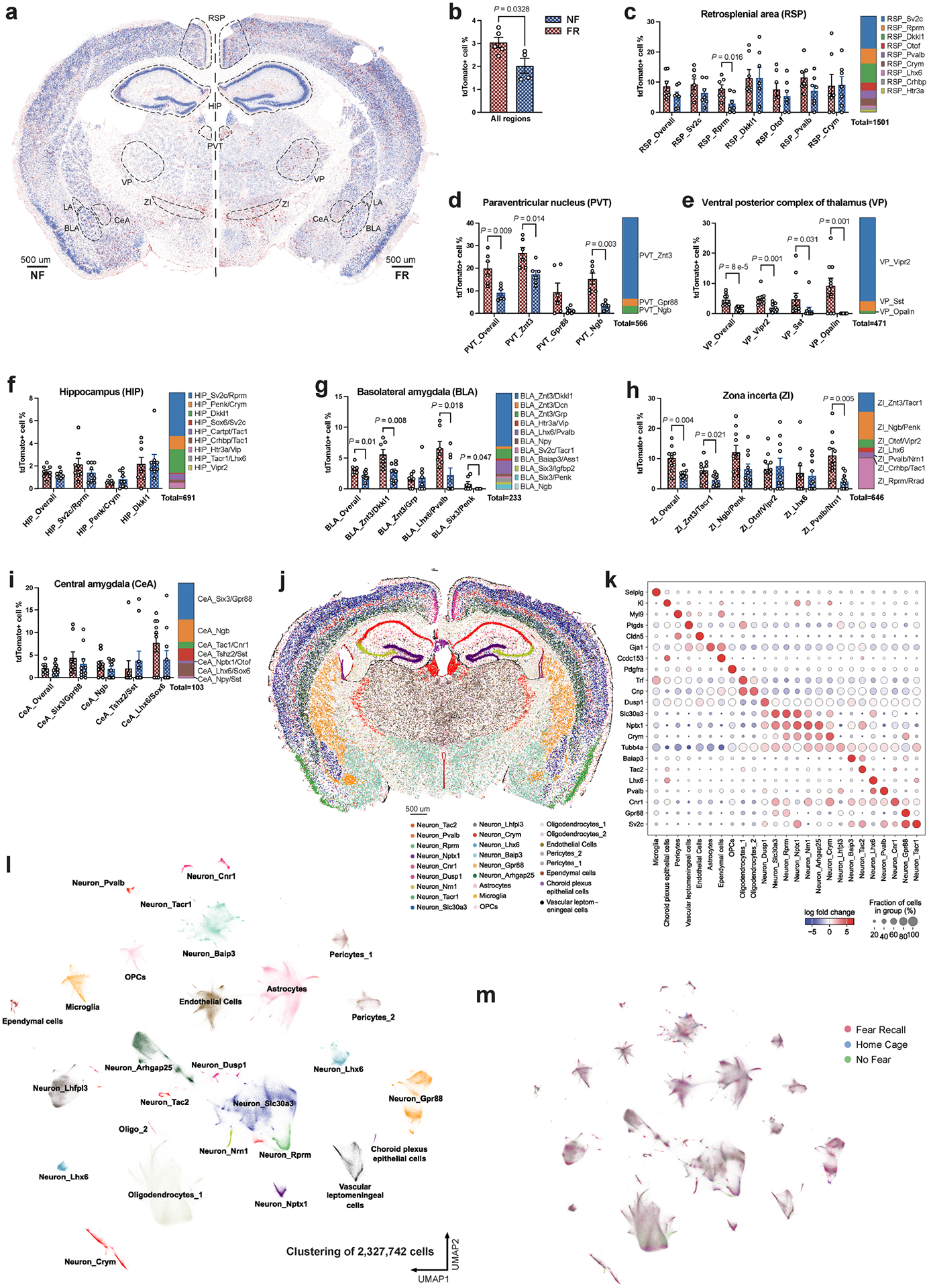
Spatial transcriptomics resolves the engram assembly in different neuronal cell types. **a)** Engram cells (tdTomato +) revealed by MERFISH. **b-i)** Quantification of tdTomato+ neurons in all regions (**b**, n _[FR]_ = 5 mice, n _[NF]_ = 4 mice), retrosplenial area (RSP, **c**, n _[FR]_ = 7 sections, n _[NF]_ = 7 sections), paraventricular nucleus of the thalamus (PVT, **d**, n _[FR]_ = 7 sections, n _[NF]_ = 6 sections), ventral posterior complex of thalamus (VP, **e**, n _[FR]_ = 10 sections, n _[NF]_ = 11 sections), hippocampus (HIP, **f**, n _[FR]_ = 8 sections, n _[NF]_ = 9 sections), basolateral amygdala (BLA, **g**, n _[FR]_ = 8 sections, n _[NF]_ = 7 sections), central amygdala (CeA, **h**, n _[FR]_ = 9 sections, n _[NF]_ = 10 sections), and zona incerta (ZI, **i**, n _[FR]_ = 10 sections, n _[NF]_ = 10 sections), mean +/− S.E.M, unpaired two-tailed student t-test. j) Unbiased clustering of all cells resolved in situ. **k)** Marker genes expression of major cell types. **l)** Major cell types with annotations resolved a UMAP. **m)** All cells grouped by HC, FR, and NF conditions. All MERFISH data.

**Extended Data Fig. 2 |:**
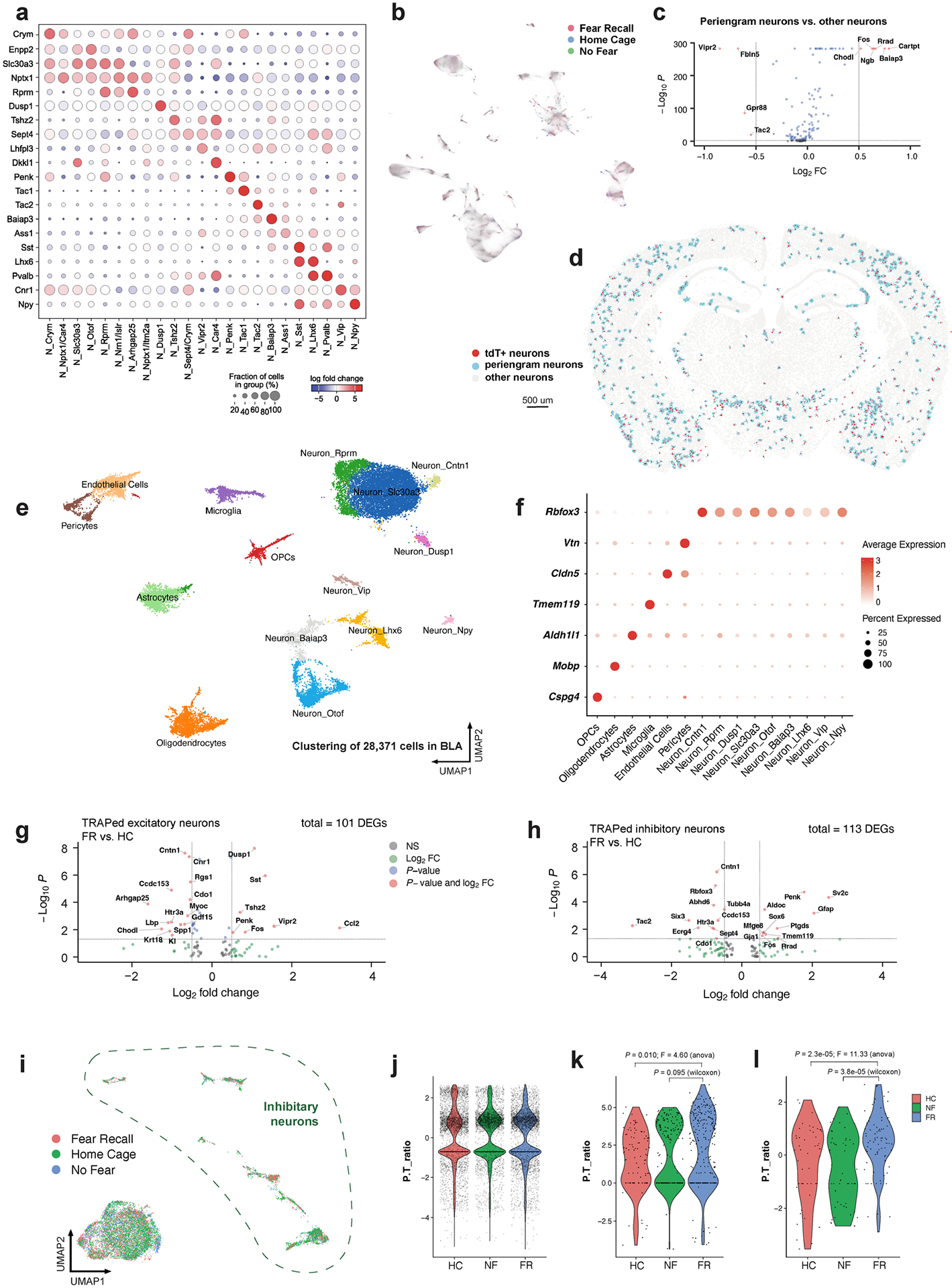
Spatial transcriptomics resolves memory associated genes. **a)** Marker genes expression of neuronal cell types. Neurons grouped by HC, FR, and NF conditions. **b)** Differentially gene expression analysis of peri-engram neurons (neurons within a radius of 30 um to engram neurons) other neurons. **c)** Genes enriched in peri-engram neurons over other neurons, unadjusted *P* value by Mann Whitney Wilcoxon test. **d)** Engram neurons and peri-engram neurons resolved in situ. **e)** Unbiased clustering of all cells from BLA. **f)** Marker genes expression of major cell types in the BLA. **g)** Fear memory induced gene expression in excitatory engram neurons of BLA, FR vs. HC. **h)** Fear memory induced gene expression in inhibitory engram neurons of BLA, FR vs. HC. **i)** BLA neurons grouped by FR and NF conditions. **j)** *Penk* to *Tac2* ratio of all neurons in BLA. **k)** *Penk* to *Tac2* ratio of TRAPed neurons in BLA, one-way ANOVA and two-sided Mann Whitney Wilcoxon test. **l)** Penk to Tac2 ratio of TRAPed inhibitory neurons in BLA, one-way ANOVA and two-sided Mann Whitney Wilcoxon test. All MERFISH data.

To visualize the gene-expression patterns of sparsely distributed engram cells, we performed spatial transcriptomic analyses with single-molecule resolution^11^, which enabled us to study TRAPed (tdT^+^) ‘engram’ cells in situ (Fig. 1c and Extended Data Fig. 1a,b). Fear memory consolidation in FR mice increased the number of tdT^+^ engram neurons compared with NF mice, especially in the BLA, paraventricular nucleus of the thalamus (PVT), ventral posterior complex of thalamus (VP) and zona incerta (ZI) (Extended Data Fig. 1c–i). The slice-based analysis we used provides spatial information and preserves the native cellular architecture of the tissue, avoiding a potential dissociation bias. Using a customized panel of 158 genes derived from scRNA-seq data, we observed 10 major classes from more than 2.3 million cells (Extended Data Fig. 1j–m), including more than 1.2 million neurons that formed at least 23 types (Fig. 1d,e and Extended Data Fig. 2a,b) and 9 major non-neuronal cell types (Extended Data Fig. 1j–m). Consistent with a previous study^12^, neurons accounted for 53.5% of the cells.

Within the BLA, we identified astrocytes, microglia, oligodendrocytes, oligodendrocyte precursor cells (OPCs), endothelial cells, pericytes (Extended Data Fig. 2e,f) and eight types of neurons (two excitatory and six inhibitory) (Fig. 1f–h). The neuron types express distinctive marker genes, including *Dkkl1* and *Tshz2* for excitatory neurons, and *Npy*, *Htr3a*, *Pvalb*, *Sst*, *Baiap3* and *Six3* for inhibitory neurons (Fig. 1f–h). Differentially activated tdT^+^ neurons in the FR condition presumably correspond to engram cells that are part of a persistent memory signature, since we are instituting the memory recall two weeks after training and are analysing gene expression after a further nine days. However, the handling of the mice in the three control conditions, especially during the NF condition, may also activate gene expression that is unrelated to memory. Because of this circumstance and possibly owing to non-specific background activation, some tdT^+^ cells are detected even in the control conditions. Therefore, we computed differentially expressed genes (DEGs) between the tdT^+^ cells in the NF and FR conditions. Since these DEGs were monitored nine days after memory recall, the DEGs are likely to represent genes whose expression is induced during the recall as a function of the previous fear memory training and are persistently expressed after being induced. Among 15,441 neurons in the BLA, 358 (3.23%) tdT^+^ neurons were identified in the FR condition versus 166 (2.06%) neurons in the NF condition (Fig. 1c). Differential gene-expression analysis in excitatory neurons identified that early response genes (*Dusp1* and *Fos*) and the neuropeptide gene *Penk* were upregulated in the FR condition over the NF condition (Fig. 1i). Genes associated with synaptic vesicles (such as *Sv2c*) and *Penk* were upregulated in the FR condition over the NF condition in inhibitory engram neurons, whereas the neuropeptide gene tachykinin 2 (*Tac2*) was down-regulated (Fig. 1j). A similar *Penk*-to-*Tac2* shift was not observed in total neurons (Extended Data Fig. 2j–l).

## A memory engram gene signature in the BLA

**Extended Data Fig. 3 |:**
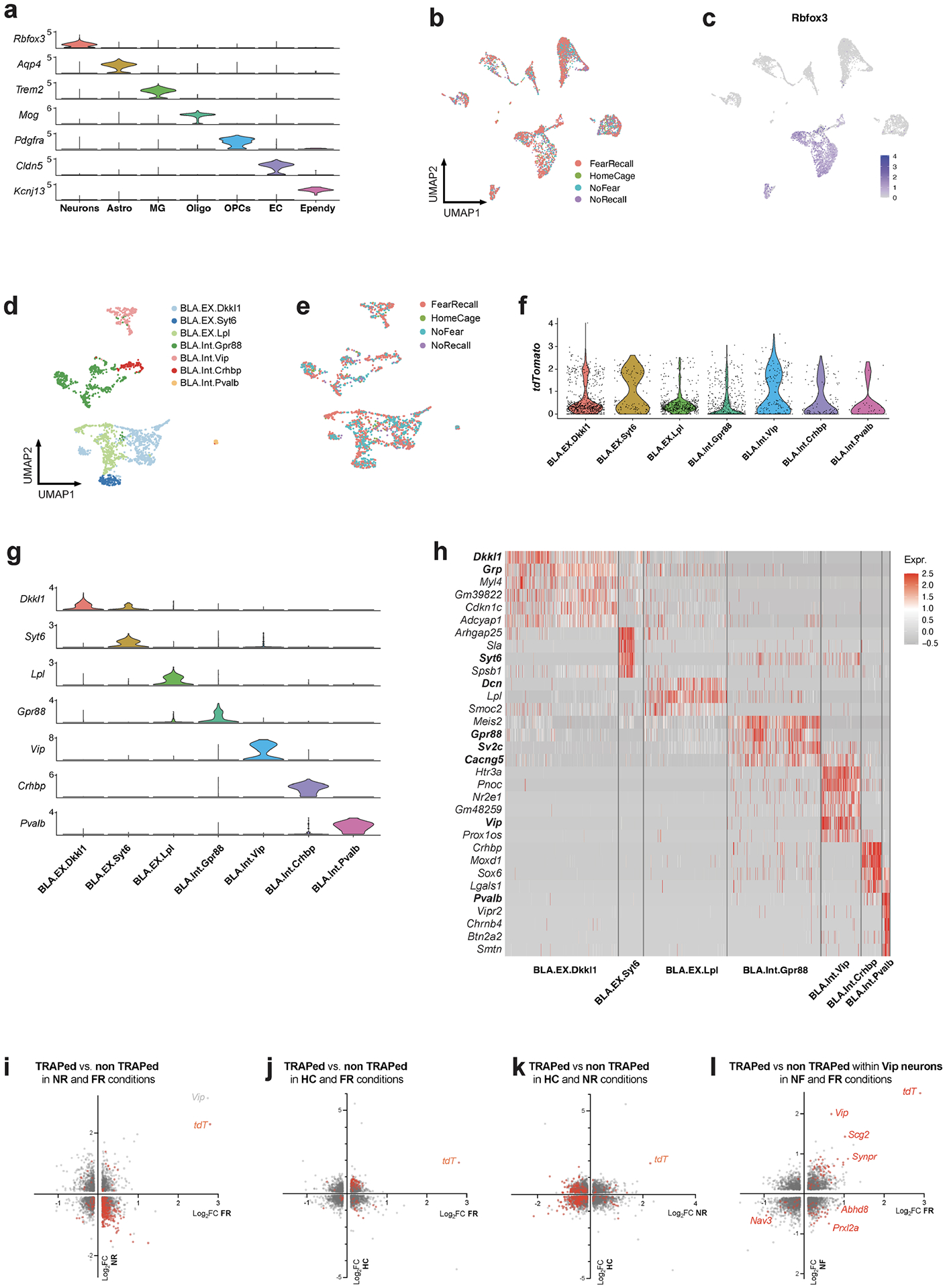
Single-cell transcriptomics resolves the engram associated genes. **a)** Distinct markers for each cluster of BLA cells. **b)** BLA cell clustering colored by training conditions. **c)** *Rbfox3* expression in BLA cells. **d)** Clustering of BLA neurons, detecting 9144 genes/cell in median. **e)** BLA neurons clustering colored by training conditions. **f)** Distinct markers for each cluster of BLA neurons. **g)** Heatmap of top marker genes of neuronal clusters **h)** tdTomato expression in each neuron cluster. **i**-**l**) DEGs of TRAPed neurons over non TRAPed neurons, red denotes significant DEGs in both conditions/axes. All scRNAseq data.

**Fig. 2 |:**
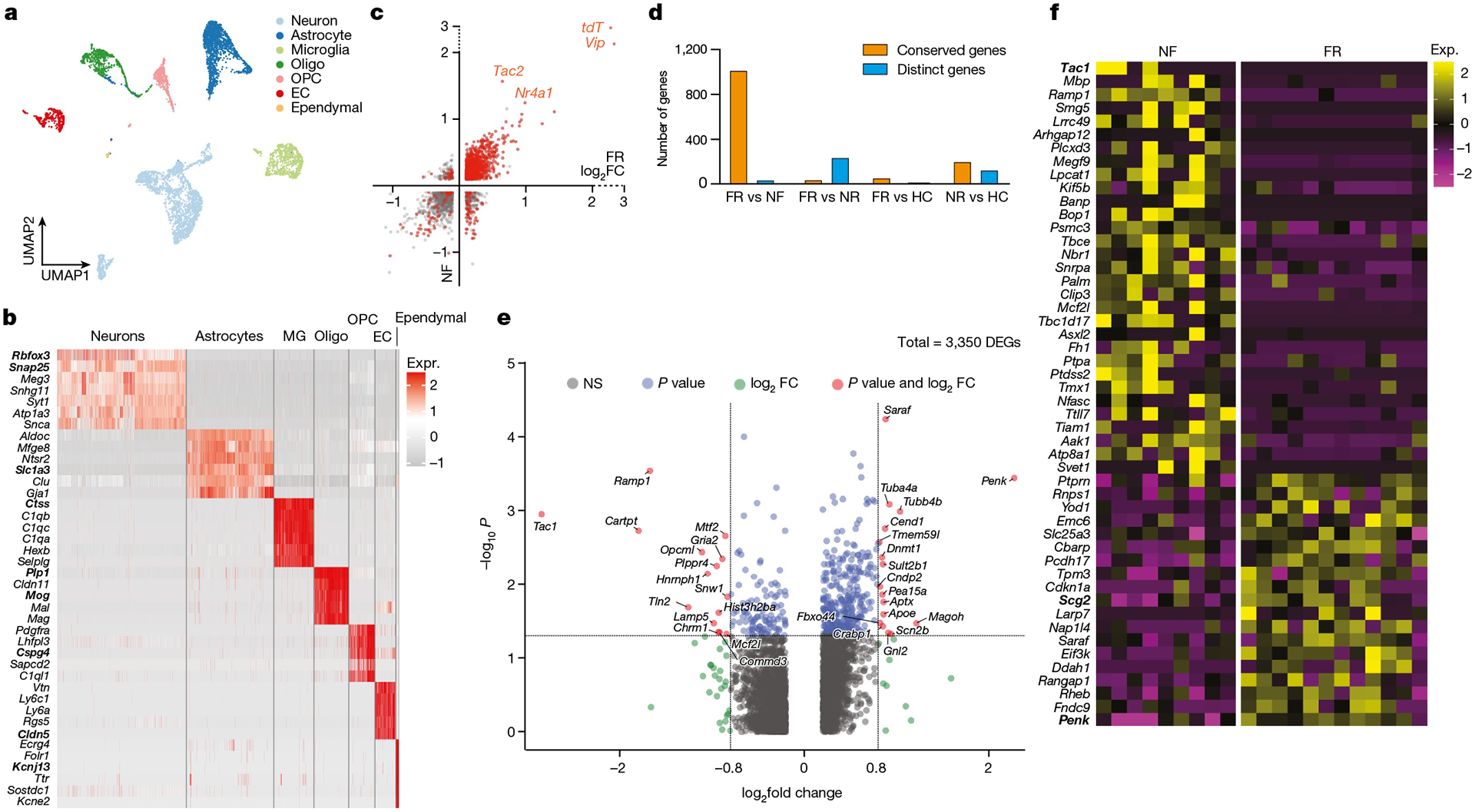
Memory consolidation evokes cell-type-specific transcriptional programmes. **a–e**, scRNA-seq data. **a**, Clustering of all cells in BLA using Smartseq3 sequencing. **b**, Distinct markers for each cluster of neurons. **c**, DEGs of TRAPed neurons over non-TRAPed neurons in the FR (*x* axis) and NF (*y* axis) condition, red denotes significant DEGs (*P* < 0.05 in both conditions (axes), two-sided Mann–Whitney–Wilcoxon test). **d**, Quantification of genes enriched in TRAPed neurons. Gene expression is mostly conserved between FR and NF, whereas genes expressed in FR and NR are mostly distinct. **e**, Volcano plot showing DEGs in FR versus NF of TRAPed BLA.Int.Gpr88 neurons, a type of P^+^T^−^ neuron. *P* < 0.05, unadjusted *P* value by Mann–Whitney–Wilcoxon test. **f**, DEGs in FR versus NF of TRAPed BlaIn.Gpr88 neurons, a type of P^+^T^−^ neuron. Each column represents one cell. EC, endothelial; MG, microglia; oligo, oligodendrocyte.

To study engram cells in depth, we used full-length deep scRNA-seq experiments^13^ with an average transcript detection of 9,144 genes per neuron. We analysed the transcriptome of 6,361 cells of the BLA, which enabled identification of all major cell types, including neurons (*Rbfox3*^+^), astrocytes (*Slc1a3*^+^), microglia (*Ctss*^+^), oligodendrocytes (*Plp1*^+^), OPCs (*Cspg4*^+^), endothelial cells (*Cldn5*^+^) and ependymal cells (*Kcnj13*^+^) (Fig. 2a,b and Extended Data Fig. 3a). Consistent with previous reports^14,15^, our scRNA-seq results are highly correlated with spatial transcriptomic analyses (Supplemental Fig. 1a–d). The relative abundance of cell types was conserved among fear memory training conditions (Extended Data Fig. 3b), suggesting that long-term fear memory formation does not alter the cellular architecture of the BLA.

Subclustering of 2,137 neurons (456 of which were tdT^+^) revealed 7 subtypes (Extended Data Fig. 3d–h) characterized by distinctive marker genes. These subtypes were consistently observed in the four training conditions (Extended Data Fig. 3e) and validated by spatial transcriptomic data (Fig. 1f,g and Extended Data Fig. 3f,g). All subtypes contained tdT^+^ cells, suggesting that all subtypes were activated during recall (Extended Data Fig. 3f).

We then analysed which genes characterize tdT^+^ cells. In addition to tdT, genes encoding neuropeptides (for example, vasoactive intestinal peptide (*Vip*) and *Tac2*) and the immediate-early gene *Nr4a1* were enriched in tdT^+^ neurons. These genes were consistently observed in both the FR and NF conditions (Fig. 2c), but not in the HC and NR conditions (Fig. 2d and Extended Data Fig. 3i–l), suggesting that the salient experience of placing the mice into the fear conditioning chamber in the NF condition is sufficient to induce a long-lasting change in gene expression. Of note, in line with our observation that *Vip* is the most prominently induced gene in tdT^+^ neurons, it has been reported that *Vip* interneurons are activated by salient cues in the BLA and that such activation is required for learning^16^. However, given that *Vip* was also induced in the NF condition, it clearly is not a fear engram gene.

## Memory-associated gene expression

**Extended Data Fig. 4 |:**
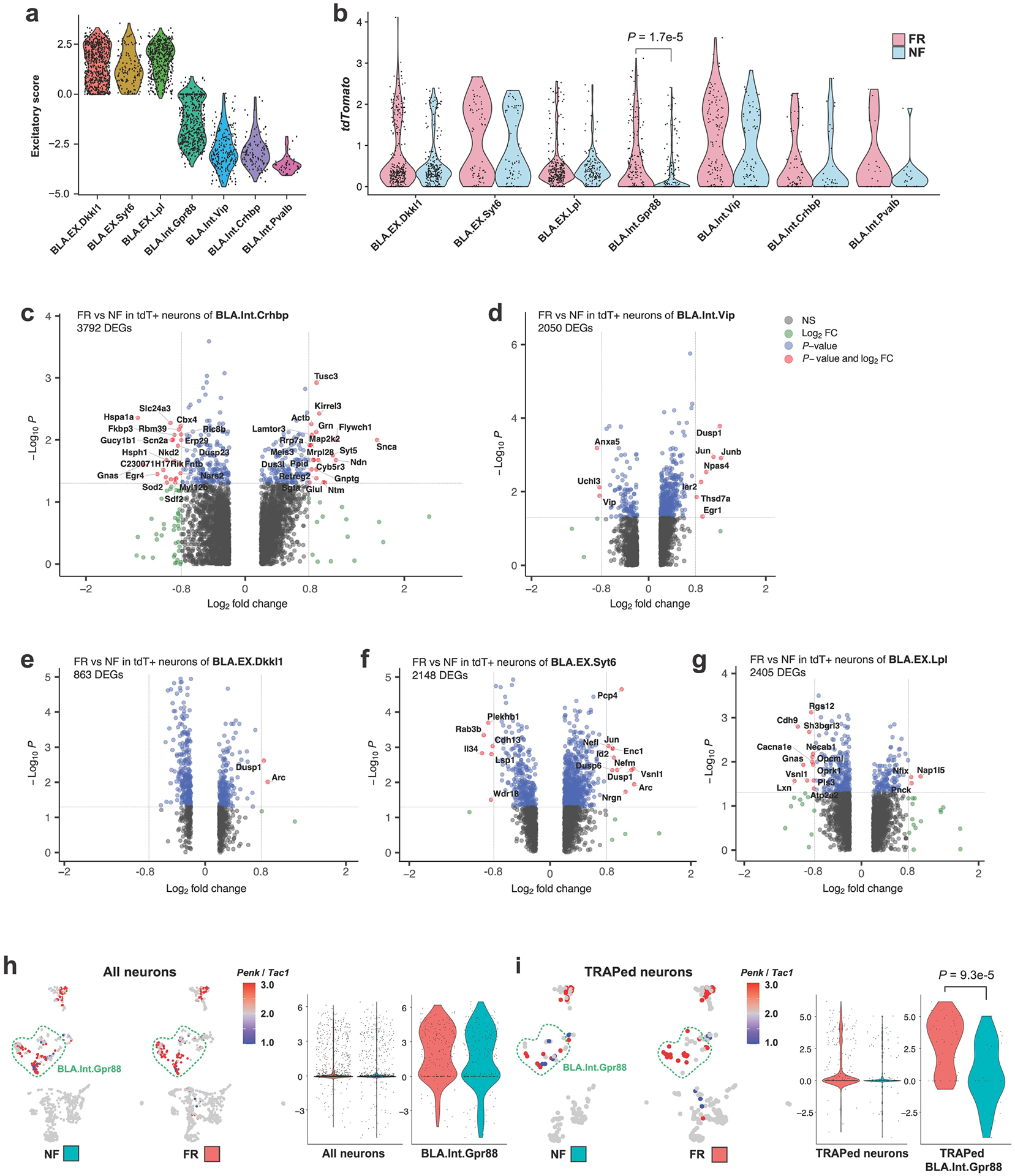
Single-cell transcriptomics resolves the memory associated genes. **a)** Excitatory score of BLA neurons, calculated by Scl17a7 – Gad1. **b)** tdTomato expression in each neuron cluster, splited by training conditions, two-tailed student T-test. **c**-**g)** DEGs of FR over NF of TRAPed BLA.Int.Crhbp (**c**), BLA.Int.Vip (**d**), BLA.EX. Dkkl1 (**e**), BLA.EX.Syt6 (**f**), and BLA.EX.Lpl (**g**) neurons, unadjusted *P* value by Mann Whitney Wilcoxon test. **i)** Penk to Tac1 ratio of all neurons in BLA. **j)** Penk to Tac1 ratio of TRAPed neurons in BLA. All scRNAseq data, *P* value calculated with Mann Whitney Wilcoxon test.

Three of the seven types of BLA neurons are glutamatergic (BLA. EX.Dkkl1, BLA.EX.Syt6 and BLA.EX.Lpl) and four are GABAergic (BLA. Int.Gpr88, BLA.Int.Vip, BLA.Int.Crhbp and BLA.Int.Pvalb) (Extended Data Fig. 4a). Notably, the FR condition recruited a significantly higher number of tdT^+^ neurons than the NF condition within the BLA.Int.Gpr88 population (Extended Data Fig. 4b), which is marked by the expression of *Gpr88*, synaptic vesicle glycoprotein 2C (*Sv2c*) and a gene encoding an AMPA-receptor associated protein (*Cacng5*) (Extended Data Fig. 3h).

To identify transcriptional changes that are specifically induced by remote memory recall in engram neurons, we screened for DEGs in TRAPed tdT^+^ neurons of FR mice versus NF mice. Single-cell resolution enables a comparison of neurons of the same type and full-length mRNA sequencing provides high-sensitivity identification of genes that are specifically associated with memory consolidation and recall. Strict criteria were applied to remove non-specific DEGs. First, DEGs that were also differentially expressed between non-TRAPed cells in FR versus NF mice were removed, which minimized the effect of basal activation. Second, only DEGs that were differentially expressed when FR cells are compared to NR and HC controls were included, ensuring that DEGs are not just a consequence of a fear experience. Finally, each DEG had to be expressed in at least one-quarter of cells and with a fold change of at least 1.75. These stringent criteria identified 107 ‘remote-memory-associated DEGs’ in 6 types of neurons (Fig. 2e and Extended Data Fig. 4c–g).

Inhibitory neurons in the BLA are known to regulate fear memory consolidation^17–19^ in a cell-type-specific manner^16,20^. Of note, the GABAergic inhibitory neurons BLA.Int.Gpr88 and BLA.Int.Crhbp exhibited more differentially expressed genes than the other neurons (Fig. 2e and Extended Data Fig. 4c–g), suggesting that inhibitory neurons in BLA are more actively involved in memory consolidation. The largest effect of remote memory recall was observed with two neuropeptide genes that were different from those detected in salience-activated gene-expression changes: *Tac1*, whose expression was suppressed more than sixfold, and *Penk*, whose expression was increased more than fourfold in BLA.Int. Gpr88 neurons (P^+^T^−^ neurons) (Fig. 2e). As a result, tdT^+^ engram neurons in the FR condition showed a much higher ratio of *Penk* to *Tac1* than tdT^+^ neurons in the NF condition in BLA.Int.Gpr88 neurons (Extended Data Fig. 4h,i). In addition, we found a strong enrichment of genes involved in MAPK pathways (*Dusp1*, *Dups6*, *Nefl*, *Lamtor3*, *Jun*, *Junb* and *Map2k2*) (Fig. 2e and Extended Data Fig. 4c–g). This result is consistent with the implication of MAPK pathways in memory consolidation in a variety of learning paradigms^21^, including fear memory consolidation in the amygdala^22^. Genes related to signalling in general, in particular BDNF signalling (*Egr1*, *Vsnl1*, *Dusp1*, *Hnrnph1*, *Id2*, *Ramp1*, *Ier2* and *Hspa1a*) (Fig. 2e and Extended Data Fig. 4c–g), were also found to be differentially regulated by fear memory. In the amygdala, BDNF signalling has been reported to be essential for fear memory consolidation^23^, fear memory extinction^24^, episodic memory formation^25^ and long-term potentiation^26^. Notably, BDNF and MAPK have been shown to relay signalling cascades and enhance stress-induced contextual fear memory^27^.

**Extended Data Fig. 5 |:**
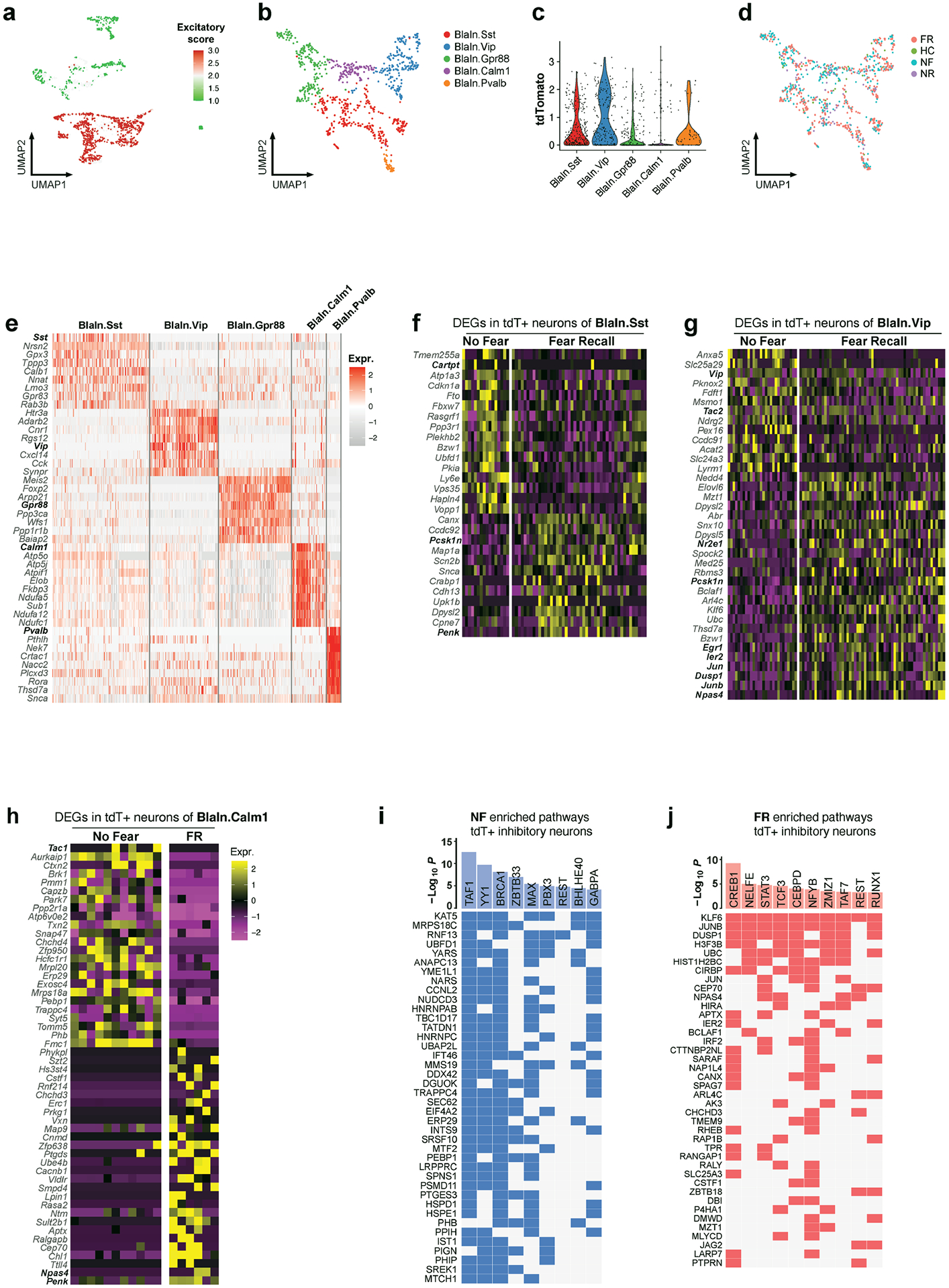
Single-cell transcriptomics resolves the memory associated genes in inhibitory neurons. **a)** Excitatory score of BLA neurons, calculated by Slc17a7 – Gad1. **b)** BLA inhibitory neuron clustering. **c)** tdTomato expression in each inhibitory neuron cluster. **d)** BLA inhibitory neuron clustering colored by training conditions. **e)** Heatmap of top marker genes of inhibitory neuronal clusters **f**-**h**) DEGs (FR over NF, TRAPed) of BlaIn.Sst (**f**), BlaIn.Vip (**g**), and BlaIn.Calm1 (**h**), each column is a cell. **i**,**j**) Transcription factor enrichment analysis of NF induced genes (**i**) or FR induced genes (**j**), unadjusted *P* value. All scRNAseq data.

Because inhibitory neurons in the BLA exhibited engram-specific gene-expression changes, we further subclustered these inhibitory neurons which revealed five subtypes: BlaIn.Sst, BlaIn.Vip, BlaIn.Gpr88, BlaIn.Calm1 and BlaIn.Pvalb (Extended Data Fig. 5a–e). Differential analysis of these TRAPed tdT^+^ inhibitory neuron subtypes between FR and NF uncovered 159 genes that were associated with memory consolidation (Fig. 2f and Extended Data Fig. 5f–h). Transcription factor enrichment analysis^28^ of the FR-induced genes revealed a strong enrichment of target genes of CREB (Extended Data Fig. 5j). The CREB signalling pathway is widely implicated in long-term memory consolidation^29^.

Although immediate-early genes (IEGs) have been widely associated with synaptic plasticity, a subset of IEGs was also regulated by long-term fear memory consolidation in addition to those induced by salience alone, including the early immediate-early response 2 (*Ier2*), early growth response 1 (*Egr1*), *Jun*, *Junb*, dual specificity phosphatase 1 (*Dusp1*) and neuronal PAS domain protein 4 (*Npas4*) genes (Fig. 2f and Extended Data Fig. 5f–h). In particular, *Egr1* was reported to be required in lateral amygdala for long-term fear memory consolidation without impairing acquisition or short-term memory^30^. *Npas4* encodes a Ca^2+^ influx-dependent protein that regulates synapse development in inhibitory neurons^28^, marks a subset of fear induced engram neurons in parallel with FOS engrams^31^, and is required for both short-term and long-term contextual fear memory^32^. Previous work in hippocampus showed that *Penk*, *Dusp1*, *CREB*, *Npas4* are also involved in fear memory^33^.

We found that several genes associated with neuropeptides were regulated during fear memory consolidation: secretogranin 2 (*Scg2*) and *Penk* were upregulated, whereas tachykinin 1 (*Tac1*) was down-regulated in BlaIn.Gpr88 neurons (P^+^T^−^ neurons) (Fig. 2f). *Scg2* was recently shown to perform an instructive role in establishing the network of Fos-activated neurons^34^. Similarly, *Penk* was upregulated in both BlaIn. Sst and BlaIn.Calm1 neurons, in which the neuropeptides cocaine- and amphetamine-regulated transcript protein (CART, encoded by *Cartpt*) and *Tac1* were down-regulated, respectively (Extended Data Fig. 5f,h). In Vip neurons, *Tac2* and *Vip* were down-regulated (Extended Data Fig. 5g). *Pcsk1n* (which encodes the neuroendocrine precursor peptidase ProSAAS) was upregulated in both BlaIn.Sst and BlaIn.Vip neurons (Extended Data Fig. 5f,g); *Pcsk1n* has been reported to control prohormone processing^35^ and to be required for fear memory^36^. Together, these data show that engram neurons switch the production of neuropeptides during memory consolidation, implicating neuropeptides as key agents in long-term memory formation. Neurons often express multiple neuropeptides that are released upon neuronal activation^37^ and stimulate diverse signalling pathways in target cells by binding to G-protein-coupled receptors to control neural activity and synaptic plasticity, processes that are essential for memory formation and emotional behaviour. It is thus plausible that neuropeptides are important in fear memory, which suggests that they might control cell signalling beyond activation of engram neurons. Moreover, more than half of the DEGs associated with remote memory have links to neuronal disorders such as dementia, mental retardation, epilepsy, schizophrenia and Charcot–Marie–Tooth disease types I and II. This indicates a potential correlation between the functional role of these genes in regulating remote memory and their involvement in the development of neurological disorders.

## The role of astrocyte remodelling

**Extended Data Fig. 6 |:**
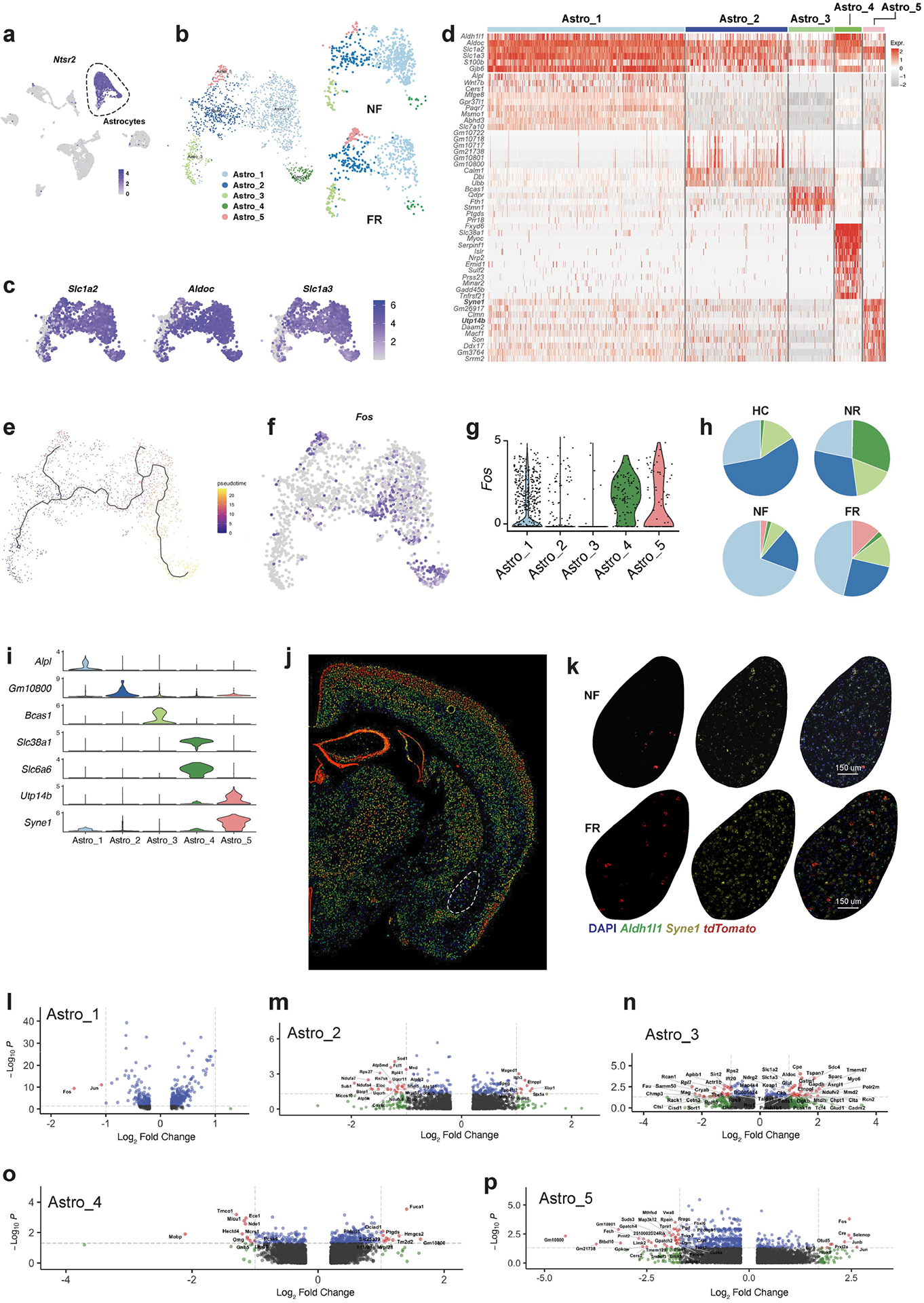
Single-cell transcriptomics resolves the memory associated genes in astrocytes. **a)** *Ntsr2* expression is enriched in astrocytes among all cells in BLA. **b)** Cluster of astrocytes from BLA. **c)** Expression level of astrocyte pan markers (*Slc1a2*, *Aldoc*, and *Slc1a3*). **d)** Heatmap of top marker genes of BLA astrocyte clusters. **e)** Cellular trajectory estimation of BLA astrocytes, based on gene expression. **f)** *Fos* expression of BLA astrocytes. **g)** *Fos* expression of astrocyte clusters **h)** Astrocyte composition separated by training conditions. **i)** Distinct markers for each astrocyte cluster from BLA. **j)** Syne1 expression data, retrieved from Allen Atlas. **k)** RNAscope in situ stanning of *Syne1* and *tdTomato* in BLA of NF and FR conditions. **l-p)** DEGs of FR vs. NF in Astro_1 – 5, unadjusted *P* value by Mann Whitney Wilcoxon test. All scRNAseq data, except **i** and **j**.

**Fig. 3 |:**
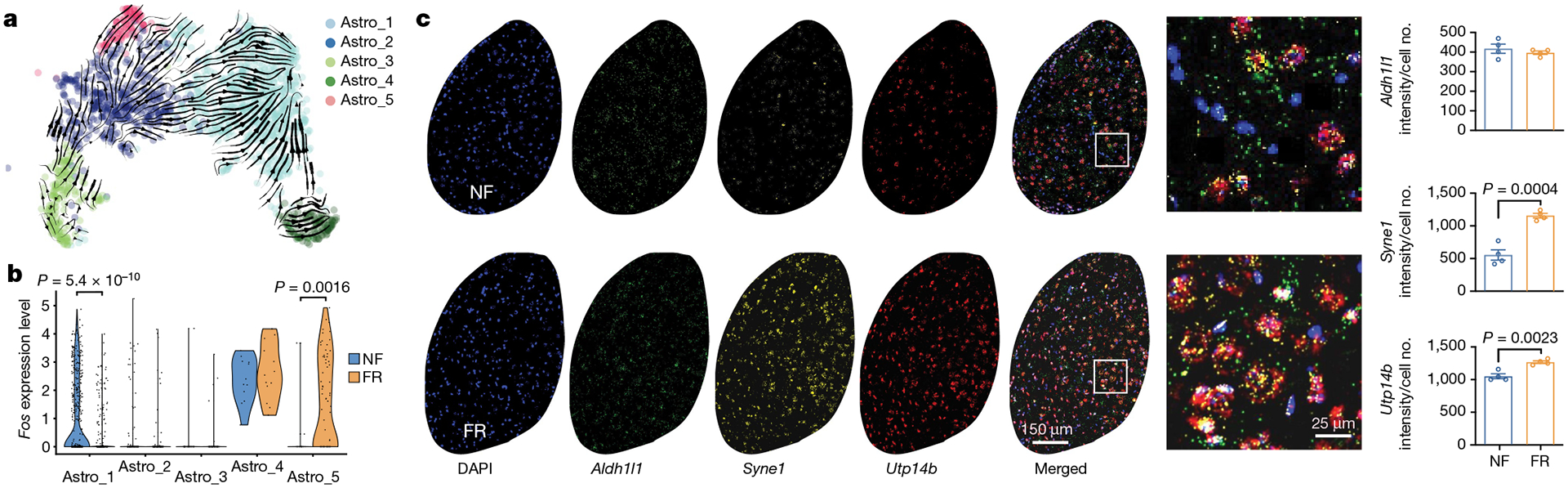
Remote memory consolidation activates astrocytes. **a**, Cellular trajectory estimation for BLA astrocytes, based on RNA maturation from scRNA-seq data. **b**, *Fos* expression of FR and NF astrocytes from scRNA-seq data. Unpaired two-tailed Student’s *t*-test. **c**, RNAscope in situ staining of *Aldh1l1*, *Syne1* and *Utp14b* transcripts in BLA of NF and FR astrocytes. *n* = 4 mice; data are mean ± s.e.m.; unpaired two-tailed Student’s *t*-test.

Neuron–glia interactions are thought to have an essential role in memory consolidation^38^. Moreover, astrocytes respond to neuronal activity with neuronal activity-dependent sharp tuning^39^. We found that neurotensin (*Nts*) is induced during memory formation in engram neurons and that the neurotensin receptor gene *Ntsr2* is primarily—and perhaps exclusively—expressed in astrocytes (Extended Data Fig. 6a), suggesting that long-term memory formation may also involve induced persistent gene-expression changes in astrocytes. Indeed, among non-neuronal cells, only astrocytes exhibited consistent transcriptional changes associated with remote memory consolidation (Fig. 3). Unbiased clustering of 1,637 astrocytes identified 5 cell states that might be considered astrocyte subtypes (Astro_1–Astro_5) (Extended Data Fig. 6b–d). Cellular trajectory analyses based on RNA dynamics^40^ and gene-expression patterns^41^ suggested a cellular pathway connecting these subtypes (Fig. 3a and Extended Data Fig. 6e).

Astrocytes engage in local interactions with neurons and other types of glia. These interactions are increasingly recognized for sensing and modulating neural circuit activity and for contributing to information processing in the brain, including memory consolidation^42,43^. Astro_4 cells, and to a lesser extent Astro_1 and Astro_5 cells, exhibited relatively high expression levels of *Fos*, suggesting that the final astrocyte cell state is an active state (Extended Data Fig. 6f,g). Of note, Astro_4 cells also express the GABA (γ-aminobutyric acid) transporter gene *Slc6a6* and the glutamine transporter gene *Slc38a1* (Extended Data Fig. 6d,i), consistent with functional roles in regulating neurotransmitter levels. Notably, memory consolidation promoted the transition from Astro_2 to Astro_5 cells and reduced the proportion of Astro_1 cells (Fig. 3a and Extended Data Fig. 6e,h). Astro_1 cells are significantly less active, whereas Astro_5 are more active, in the FR than in the NF condition suggesting that memory consolidation shifted active astrocytes from the Astro_1 to the Astro_5 state (Fig. 3b). A recent study using in vivo fibre photometry showed that astrocytes in the BLA are activated during acquisition and expression of contextual fear memory^44^, consistent with our observation that the astrocyte cell types are remodelled by fear memory consolidation (Fig. 3a and Extended Data Fig. 6e–h).

The expression of *Syne1* in astrocytes is relatively low in BLA under basal conditions (Fig. 3c and Extended Data Fig. 6j,k) but was induced in the FR condition (Fig. 3c and Extended Data Fig. 6i,k). In agreement with the scRNA-seq data, we also found in the spatial transcriptomics data a subcluster of astrocytes that were induced by the FR condition (Extended Data Fig. 6a–d). This subcluster expresses high levels of *Syne1*, *Utp14b* and *Flt1* (Extended Data Fig. 6d). Notably, *Flt1* is a vascular endothelial growth factor receptor that is expressed in activated astrocytes^45^, may induce angiogenesis^45^, and could facilitate synaptogenesis^46^. An astrocyte subtype marked by *SON*, *MACF1* and *SYNE1* was recently identified in the human anterior cingulate cortex^47^. *Utp14b* was found to be upregulated in astrocytes of the neocortex in stressed mice^48^. Humans with *Syne1* mutant are more likely to develop autism^49^ and bipolar disorder^50^. In activated Astro_4 cells in the FR condition, glutathione-independent prostaglandin D synthase (*Ptgds*) and mitochondrial glutathione transporter (*Slc25a39*) genes were significantly induced (Extended Data Fig. 6o), suggesting that prostaglandin D may also be involved in memory consolidation. Meanwhile genes associated with glutamate transport (*Slc1a2* and *Slc1a3*) and glutamine synthesis (*Glul*) were upregulated in FR compared with NF in Astro_3 cells (Extended Data Fig. 6n).

**Extended Data Fig. 7 |:**
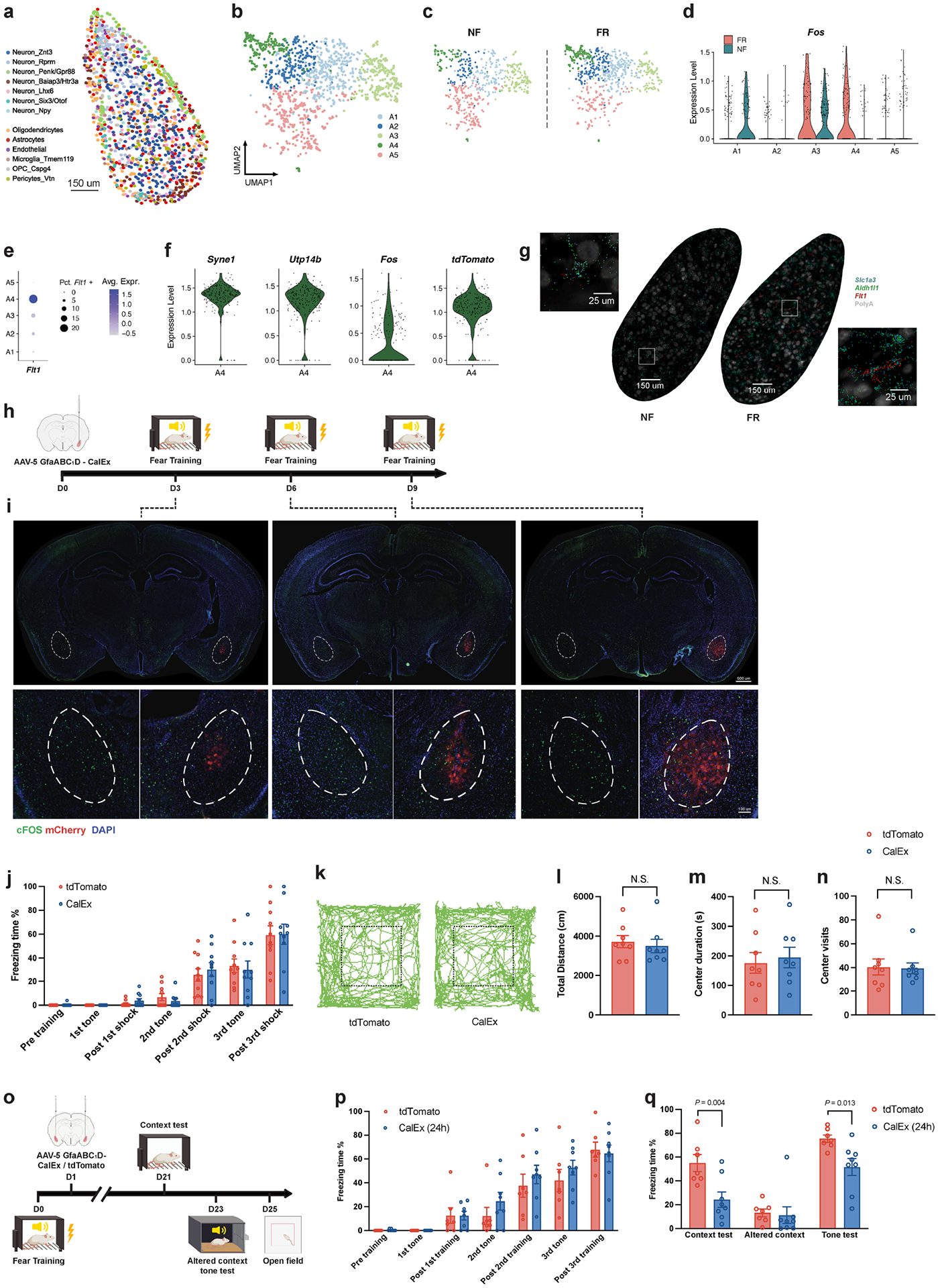
Spatial transcriptomics resolves the memory associated genes in astrocytes. **a)** Spatial embedding of all BLA cell types from MERFISH data. **b)** Clustering of astrocytes in BLA from MERFISH data. **c)** Clustering of astrocytes in BLA from MERFISH data, separated by training conditions. **d)** *Fos* expression in BLA astrocyte subtypes separated by conditions. **e)** *Flt1* expression in BLA astrocyte subtypes from MERFISH data. **f)** *Syne1*, *Utp14b*, *Fos*, *tdTomato* level in A4 astrocytes from BLA in FR, MERFISH data. **g)** *Slc1a3*, *Aldh1l1*, and *Flt1* in situ data from MERFISH. **h)** Scheme, adeno-associated virus conveying GfaABC_1_D-mCherry-CalEx were unilaterally injected to BLA C57B/6 mice. Mice were subjected to fear conditioning training at time indicated in the scheme. **i)** Immunostaining of Fos and mCherry in animals injected with GfaABC_1_D-mCherry-CalEx, n_[d3]_ = 4 mice, n_[d6]_ = 3 mice, n_[d9]_ = 4 mice. **j)** Freezing time in training, n = 8 mice, average +/− SEM. **k)** Representative tracks in open field test. **l**-**n**) Total distance (**l**), center visits (**m**), and center duration (**n**) in open field test, n = 8 mice, average +/− SEM, two-tailed student T-test. **o)** Scheme, adeno-associated virus conveying GfaABC_1_D-mCherry-CalEx (or GfaABC_1_ D-mCherry) were bilaterally injected to BLA C57B/6 mice, 24 h after fear conditioning training. Mice were subjected to context test, altered context tone test, and open field test at time indicated in the scheme. **p)** Freezing time in training, n _[tdTomato]_ = 7 mice, n _[CalEX 24h]_ = 8 mice, average +/− SEM. **q)** Mice with CalEx showed reduced freezing than tdTomato control group in context test and altered context but reduced freezing in tone test, n _[tdTomato]_ = 7 mice, n _[CalEX 24h]_ = 8 mice, mean +/− S.E.M, two tailed student T-test. **a**-**g** are MERFISH data.

**Fig. 4 |:**
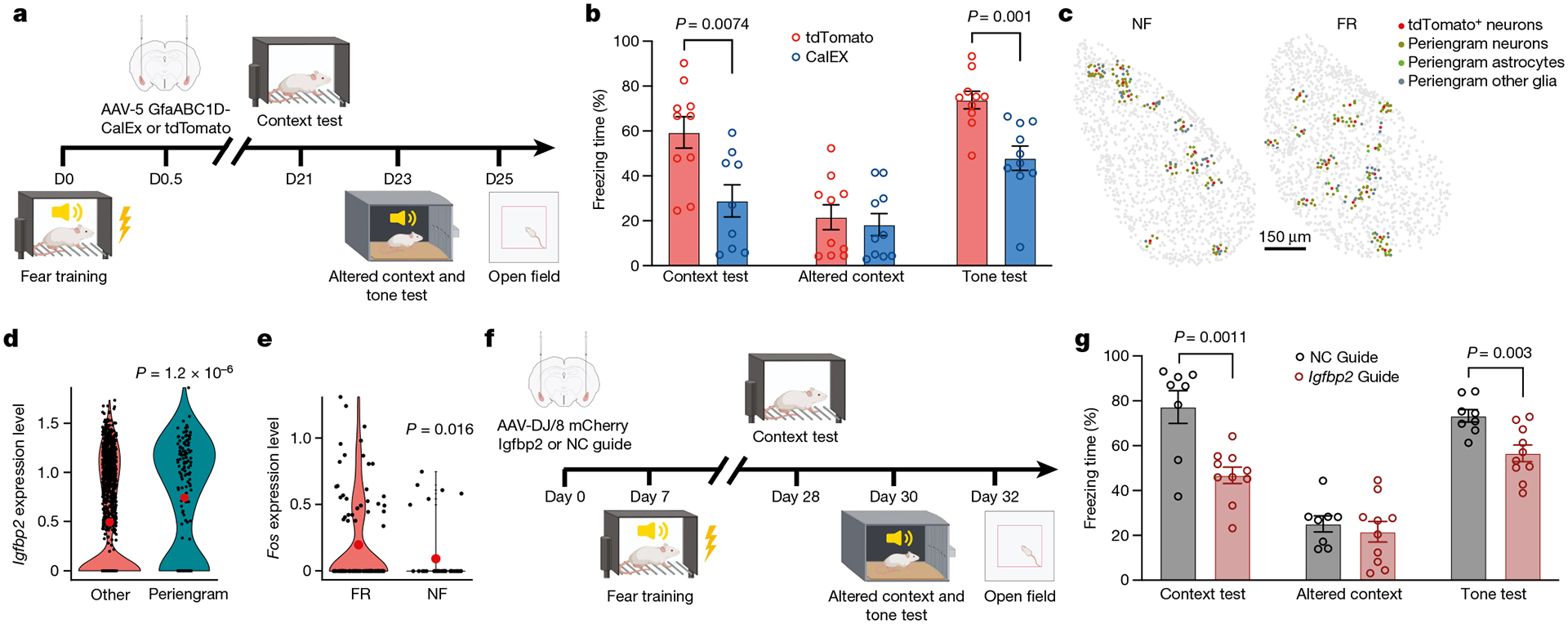
Astrocytic activation modulates long term memory consolidation. **a**, Experimental scheme. AAV expression constructs GfaABC_1_D-mCherry-CalEx (or GfaABC_1_D-tdTomato) were injected bilaterally into BLA C57B/6 mice 12 h after fear conditioning training. Mice were subjected to the context test, altered context tone test and open field test at the indicated times. **b**, Mice expressing CalEx exhibited reduced freezing compared with the tdT control group in the context test (tdTomato: *n* = 10 mice, CalEx: *n* = 9 mice), both groups exhibited comparable freezing in altered context but CalEx showed reduced freezing in the tone test than tdT control group (*n* = 10 mice). Data are mean ± s.e.m.; two-tailed Student’s *t*-test. **c**, MERFISH analysis shows spatially resolved peri-engram cells surrounding tdT^+^ neurons. **d**, *Igfbp2* expression is enriched in astrocytes surrounding tdT^+^ neurons. MERFISH data; two-sided Mann–Whitney–Wilcoxon test. **e**, Analysis of MERFISH data shows that *Fos* expression is induced in peri-engram astrocytes in the FR condition relative to the NF condition. Two-sided Mann–Whitney–Wilcoxon test. **f**, Experimental scheme. AAV constructs for expression of U6-Igfbp2 guide RNA (gRNA) (or U6-negative control (NC) gRNA) were bilaterally injected to CAG-Cas9 mice, seven days before fear conditioning training. Mice were subjected to the context test, altered context tone test and open field test at the indicated times. **g**, Mice expressing *Igfbp2* gRNA showed reduced freezing compared with the control group in the context test, altered context test and reduced freezing in the tone test. NC gRNA: *n* = 8 mice, *Igfbp2* gRNA: *n* = 10 mice; data are mean ± s.e.m.; unpaired two-tailed Student’s *t*-test.

To functionally assess whether activation of astrocytes contributes to memory formation, we selectively inhibited astrocyte activation in the BLA during fear memory formation using expression of the plasma membrane Ca^2+^ ATPase CalEx, which removes calcium from astrocytes^51^ (Fig. 4a,b). After fear conditioning training, mice were bilaterally injected with adeno-associated viruses (AAVs) expressing CalEx under control of the astrocyte-specific GfaABC_1_D promoter, using mCherry as a marker and tdTomato-only expression as a control. Twenty-one days later, mice were subjected to contextual memory tests in the original and then an altered context, followed by a cued fear conditioning test and open field measurements (Fig. 4a). We found that both contextual and cued fear conditioning memory were impaired by the suppression of astrocyte activation, whereas the response to the altered context remained unchanged and low (Fig. 4b). No change in the open field test was detected (Extended Data Fig. 7k–n). Previous studies have shown that activating astrocytes in the BLA promotes fear memory formation^52^, that activating CA1 astrocytes enhances memory allocation with increased neuronal activity in learning^53^, that astrocyte activation in the hippocampus is required for long-term memory^54^, and that CA1 astrocyte activation is involved in encoding reward location^55^. This evidence further supports the notion that the activity of astrocytes is functionally linked to memory formation.

## Astrocyte–neuron interactions

**Extended Data Fig. 8 |:**
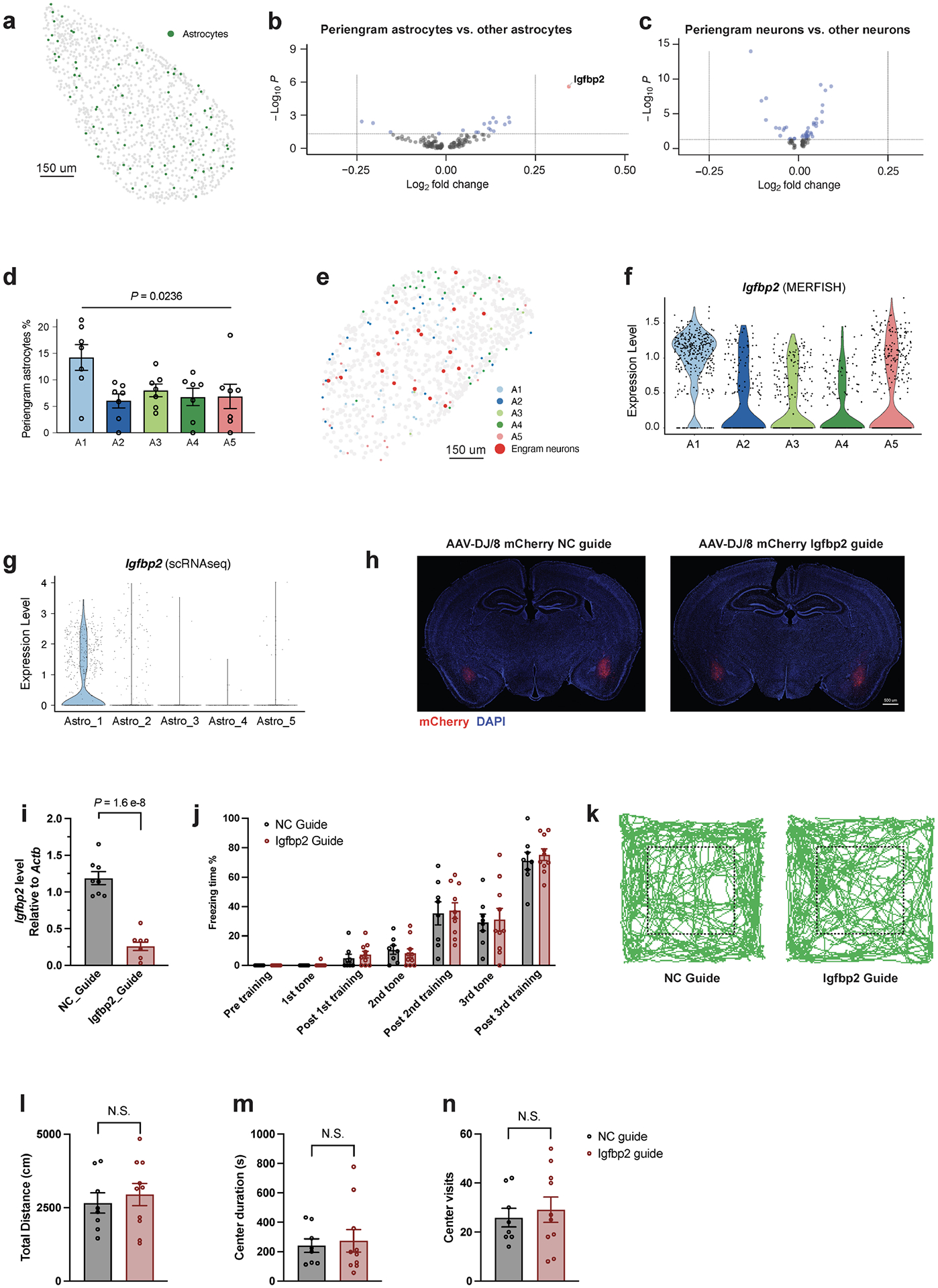
Spatial transcriptomics resolves the memory associated genes in periengram astrocytes. **a)** Spatial distribution of astrocytes in BLA. **b)** Genes differentially expressed in peri-engram astrocytes in BLA in a Volcano plot, unadjusted *P* value by Mann Whitney Wilcoxon test. **c)** Genes differentially expressed in peri-engram neurons in BLA in a Volcano plot, unadjusted *P* value by Mann Whitney Wilcoxon test. **d)** Peri-engram astrocytes percentage in each astrocyte population, n = 7 sections, on-way ANOVA test, F _(4, 30)_ = 3.296. **e)** *Igfbp2* expression in each astrocyte population in BLA, MERFISH. **f)** Spatial distribution of astrocytes and engram neurons in BLA. **g)** *Igfbp2* expression in each astrocyte population, scRNAseq data. **h)** Immunostaining of mCherry in animals injected with AAV convey *Igfbp2* guide RNA or negative control guide RNA, n = 4 mice. **i)** Relative level of *Igfbp2* RNA in BLA of animals with guide RNA injection, n _[NC guide]_ = 8 mice, n _[Igfbp2 guide]_ = 7 mice, mean +/− S.E.M, unpaired two-tailed student t-test. **j)** Freezing time in training, n _[NC guide]_ = 8 mice, n _[Igfbp2 guide]_ = 10 mice, mean +/− SEM. **k)** Representative tracks in open field test. **l**-**n**) Total distance (**l**), center visits (**m**), and center duration (**n**) in open field test, n _[NC guide]_ = 8 mice, n _[Igfbp2 guide]_ = 10 mice, average +/− SEM, two-tailed student T-test. **a**-**f** are MERFISH data.

**Extended Data Fig. 9 |:**
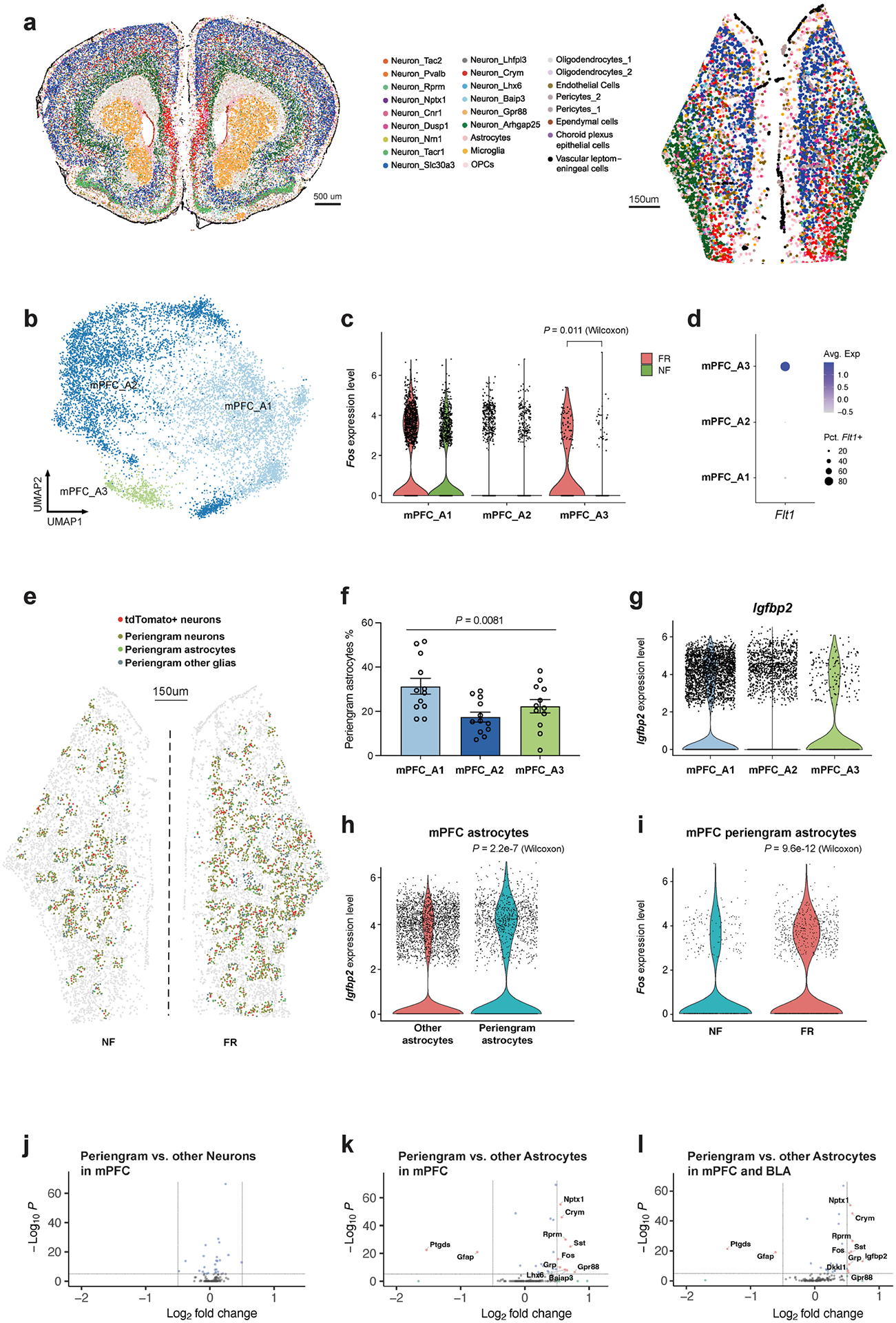
Spatial transcriptomics resolves the memory associated genes in astrocytes of mPFC. **a)** Spatial embedding of all mPFC cell types from MERFISH data. **b)** Clustering of astrocytes in mPFC from MERFISH data. **c)** *Fos* expression in mPFC astrocyte subtypes separated by conditions, two-sided Mann Whitney Wilcoxon test. **d)** *Flt1* expression in mPFC astrocyte subtypes from MERFISH data. **e)** Spatial resolved peri-engram cells surrounding tdT+ neurons in mPFC, MERFISH data. **f)** Peri-engram astrocytes percentage in each astrocyte population, one-way ANOVA F (2, 33) = 5.598, n = 12 mice. **g)** *Igfbp2* expression in each astrocyte population in mPFC, MERFISH **h)** *Igfbp2* expression is enriched in peri-engram astrocytes in mPFC (Mann Whitney Wilcoxon test, MERFISH data). **i)** *Fos* expression is enriched in FR condition than NF condition among peri-engram astrocytes in mPFC (Mann Whitney Wilcoxon test, MERFISH data). **j)** Genes differentially expressed in peri-engram neurons in mPFC in a Volcano plot, unadjusted *P* value by Mann Whitney Wilcoxon test. **k)** Genes differentially expressed in peri-engram astrocytes in mPFC in a Volcano plot, unadjusted *P* value by Mann Whitney Wilcoxon test. **l)** Genes differentially expressed in peri-engram astrocytes in mPFC and BLA, unadjusted *P* value by Mann Whitney Wilcoxon test. All MERFISH data.

Engram neurons are thought to be randomly distributed in the amygdala and other brain regions. However, some spatial structures are critical for memory formation and retrieval. Perineuronal nets are thought to modulate neuronal electrical activity by acting as a physical barrier. Perineuronal nets have been proposed to be the code book of long-term memory^56^, to be required for memory precision by allocating sparse engram cells^57^, and to contribute to memory stabilization for remote retrieval^58^. We tested whether a particular spatial cellular environment may be associated with engram neurons. By analysing the cells surrounding tdT^+^ neurons in the BLA (within a radius of 30 μm) (Fig. 4c), we detected enriched expression of *Igfbp2* (encoding insulin-like growth factor binding protein 2) in peri-engram astrocytes, whereas gene-expression patterns in peri-engram neurons were indistinguishable from those of other neurons (Fig. 4d and Extended Data Fig. 8a–c). IGFBP2, an astrocytic secreted protein, has multiple effects on neurons, including changes in synaptic transmission and excitability^59^. We found that peri-engram astrocytes are more likely to be A1 astrocytes among A1–5 BLA astrocytes revealed by MERFISH (Extended Data Figs. 6b and 8d) and the expression of *Igfbp2* is enriched in A1 and Astro_1 astrocytes (Extended data Fig. 8f,g). We found that peri-engram astrocytes exhibited a higher *Fos* activation in the FR than in the NF condition (Fig. 4e). Consistent with this finding in the BLA, we observed that *Igfbp2* is also enriched in the peri-engram astrocytes in medial prefrontal cortex (mPFC) (Extended Data Fig. 9a–l). Our spatial transcriptomic data not only localized the sparse engram cells and identified the signatures of cells in close vicinity to engram cells, but also recapitulated the scRNA-seq-defined cellular structure and gene expression of engram, and the activation of astrocytes by memory consolidation.

To ascertain the functional significance of the increased expression of *Igfbp2* in peri-engram astrocytes for memory formation, we deleted *Igfbp2* in the BLA prior to fear memory induction. We bilaterally injected AAVs encoding a control or an *Igfbp2*-specific gRNA into the BLA of mice expressing Cas9 seven days before fear training (Fig. 4f). Three weeks post-training, we assessed these mice in contextual memory paradigms, using both the original and an altered context, followed by a cued fear conditioning test and an open field test (Fig. 4f). The *Igfbp2* knockout in the BLA led to pronounced deficits in both contextual and cued fear conditioning (Fig. 4g). However, responses to the altered context remained consistently low and unchanged (Fig. 4g). The open field test did not indicate any behavioural alterations following the deletion of *Igfbp2* in the BLA (Extended Data Fig. 8k–n). Of note, a recent study demonstrated that a peptide derived from *Igfbp2* could enhance neuroplasticity and ameliorate the phenotypic deficits observed in a mouse model of Phelan–McDermid syndrome^60^.

## A memory link between PFC and amygdala

**Extended Data Fig. 10 |:**
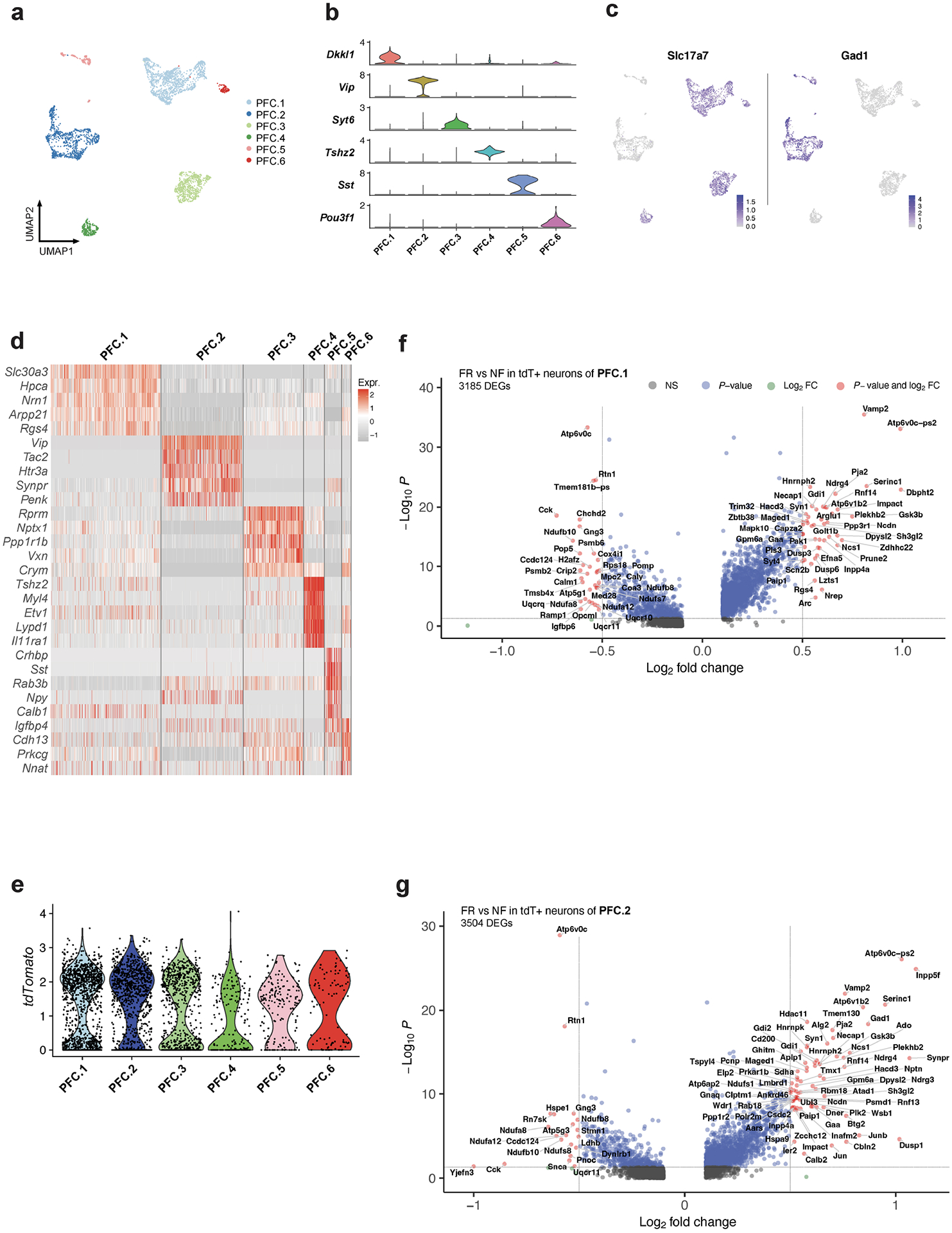
(reanalysis of scRNAseq data of mPFC neurons, Chen et al., 2020) Single-cell transcriptomics resolves the memory associated genes in mPFC. **a)** Cluster of mPFC neurons **b)** Distinct markers for each cluster of mPFC neurons. **c)** *Slc17a7* and *Gad1* expression of mPFC neurons. **d)** Heatmap of top marker genes of mPFC neurons. **e)** tdTomato expression of mPFC neurons. **f)** DEGs of TRAPed cells from PFC.1, unadjusted *P* value by Mann Whitney Wilcoxon test. **g)** DEGs of TRAPed cells from PFC.2, unadjusted *P* value by Mann Whitney Wilcoxon test. All scRNAseq data.

Although fear memory formation is orchestrated by the convergent contributions of many brain regions, the mPFC and amygdala are recognized as a key signalling axis in the process. We used our earlier deep scRNA-seq data from the mPFC^9^ (Extended Data Fig. 10a–g) for an integrated analysis of neurons from the BLA and mPFC to test whether a common gene-expression signature connects long-term memory formation in these two regions.

**Fig. 5 |:**
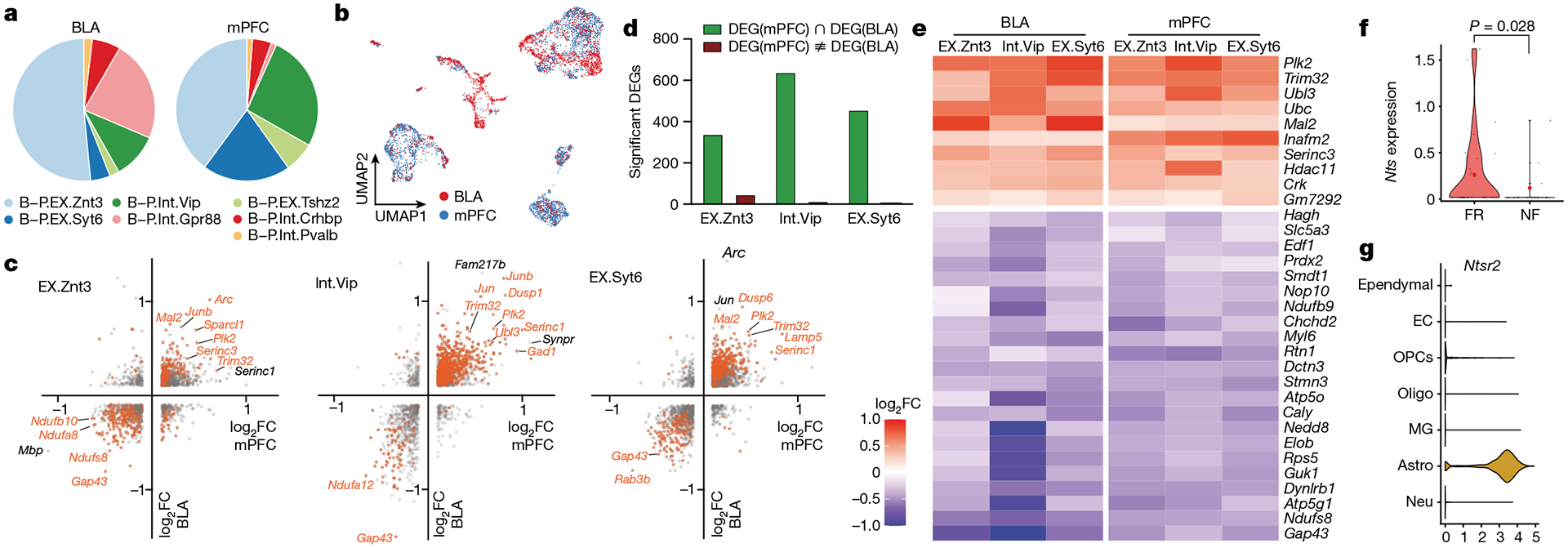
Engram neurons in mPFC and BLA share transcriptional machinery in consolidating remote memory. **a**–**g**, Analysis of scRNA-seq data. **a**, Cellular composition of BLA and mPFC. **b**, Integrated clustering of BLA and mPFC neurons, coloured by region. **c**, DEGs of TRAPed cells of EX.Znt3 (left), Int.Vip (middle) and EXT.Syt6 (right). The *x* axis shows fold change of FR over NF in BLA and the *y* axis shows the fold change of mPFC. Significant DEGs are shown in orange. *P* < 0.05 for both conditions (axes); two-sided Mann–Whitney–Wilcoxon test. **d**, Quantification of significant DEGs in neuron clusters 1–3. **e**, DEGs (FR over NF, TRAPed cells) from BLA and mPFC among B-P.EX.Znt3, B-P. Int.Vip, and B-P.EX.Syt6 neurons. **f**, *Nts* expression in tdT^+^ B-P.EX.Syt6 neurons from BLA. Two-sided Mann–Whitney–Wilcoxon test. **g**, *Ntsr2* expression in all cells from BLA, *Ntsr2* expression is highly enriched in astrocytes.

Deep scRNA-seq data from a set of 4,603 neurons from the mPFC and BLA were used to cluster the neurons into 7 populations with clear markers for each cell type (Extended Data Fig. 12a–d). Of note, six of the seven types of neurons were found in both the BLA and mPFC; only Gpr88 neurons were specific to the BLA (Fig. 5a,b and Extended Data Fig. 12b). EX.Znt3, EX.Syt6 and EX.Tshz2 cells are excitatory neurons that express the vesicular glutamate transporter 2 (vGlut2) gene (*Slc17a7*), whereas the other clusters are Gad1^+^ inhibitory neurons (Extended Data Fig. 12c). Among all neuron types, relatively more TRAPed tdT^+^ neurons were found in the mPFC than the BLA (Extended Data Fig. 12e).

Next, we examined the FR-induced transcriptional changes within the TRAPed neurons of each type of neuron. Integrated differential expression analysis identified 1,673 genes that were significantly changed in both the BLA and mPFC (Fig. 5c and Extended Data Fig. 12f). Unexpectedly, 1,587 (94.9%) of the DEGs were co-regulated in the same direction (Fig. 5d). This suggests that memory consolidation drives a conserved transcriptional programme in engram neurons across multiple brain regions. Consistent with the above analyses, DEGs associated with vesicle exocytosis and synapse formation were upregulated. Furthermore, within the engram cells of the three most abundant neuron types (EX.Znt3, Int.Vip and EX.Syt6) we found 32 genes whose expression was consistently modulated by long-term fear memory in both the BLA and mPFC (Fig. 5e). Among the top upregulated genes, Polo-like kinase 2 (*Plk2*) is a transcriptional target of NPAS4 that modulates synapse formation and contextual fear memory^61^, and *Trim32* (which encodes the E3 ubiquitin ligase TRIM32), *Ubl3* and *Ubc* are involved in protein ubiquitination, which is involved in synaptic plasticity^62^ and fear memory formation in the hippocampus^63^ and amygdala^64^. *Mal2* encodes an integral membrane constituent of synaptic vesicles associated with vGlut1-positive nerve terminals^65^. These data suggest that engram neurons in the prefrontal cortex (PFC) and BLA share overlapping transcriptional signatures mediating memory consolidation.

## Conserved neuron-to-astrocyte interaction

In addition to these conserved mechanisms, we found that neurotensin, which modulates associative memory in the paraventricular thalamus-to-BLA circuit^66^, was expressed in TRAPed Syt6-positive excitatory neurons of both the mPFC and the BLA (Extended Data Fig. 12h) and induced by fear memory consolidation in BLA but not in PFC engram neurons (Fig. 5f and Extended Data Fig. 12i). This further validates the notion that neuropeptides, including neurotensin, secretogranin, tachykinin, proenkephalin, ProSAAS and CART, are involved in memory consolidation in BLA engram cells. In addition, neurotensin receptor 2 (*Ntsr2*) is dominantly expressed by astrocytes in the BLA (Fig. 5g), whereas neurotensin receptor 1 (*Ntsr1*) is virtually undetectable in the BLA (Extended Data Fig. 12j). *Ntsr2* is essential for contextual fear memory^67^. The bidirectional communications between neurons and astrocytes are intricate and exhibit both cell-type-specific and circuit-specific characteristics^68^. Our data substantiate this dynamic by revealing that engram neurons in the BLA engage in multifaceted interactions with astrocytes during the process of memory consolidation. In particular, *Igfbp2* released from peri-engram astrocytes influences neurons, whereas neurotensin secreted by neurons acts on astrocytes during memory consolidation.

An atlas of astrocytes across brain regions has demonstrated the molecular heterogeneity of astrocytes^69^. To further understand astrocyte remodelling in memory consolidation, we clustered the integrated data from 2,278 BLA and mPFC astrocytes into four subtypes, in which B-P.A1 cells express thyroid hormone transporter (*Slco1c1*) and amino acid transporter (*Slc7a10*), B-P.A2 and B-P.A3 cells express calmodulin 1 (*Calm1*) and sphingosine-1-phosphate receptor 1 (*S1pr1*), B-P.A4 cells express myocilin (*Myoc*) and *Vim*, and B-P.A5 cells express synaptic nuclear envelope protein 1 (*Syne1*), SON DNA and RNA binding protein (*Son*) and *Utp14b* (Extended Data Fig. 13a–f). B-P.A1, B-P.A2, B-P.A3 and B-P.A4 astrocytes were present in the mPFC and BLA, whereas B-P.A5 astrocytes were specific to BLA (Extended Data Fig. 13a,g). Fear conditioning remodelled the distribution of astrocyte subtypes, in which fear recall induced B-P.A5 in BLA and B-P.A1 in the mPFC (Extended Data Fig. 13g). Of interest, astrocytes from all training conditions in both the mPFC and BLA exhibited consistent *Fos* expression in B-P. A4, but varied *Fos* expression in B-P.A1 and B-P.A5 (Extended Data Fig. 13b). These active B-P.A4 astrocytes encompass the majority of Astro_4 cells derived from the BLA (Extended Data Fig. 13a), exhibit unique expression of *Fxyd6* (which encodes FXYD domain containing ion transport regulator 6)*, Ass1* (which encodes argininosuccinate synthetase), *Slc6a6* and *Slc38a1*, which suggests a potential role of these astrocytes in modulating ion balance as well as scavenging and synthesis of neuronal transmitters in both mPFC and BLA (Extended Data Fig. 13d).

## Summary

Upon acquisition, information is initially stored as recent memory and becomes long-term memory through consolidation. Using activity-dependent cell trapping, spatial and single-cell transcriptomics, and in vivo perturbations, we identified: (1) a memory-induced activating trajectory of astrocytes; (2) a persistent gene-expression programme induced by memory consolidation, independent of salient experience, that involves neuropeptide signalling, the MAPK pathway, CREB-mediated gene expression, BDNF signalling and genes mediating neuronal synapse assembly; (3) fear memory-induced *Penk* and reduced *Tac1* expression in BLA specific Gpr88^+^ neurons; and (4) a spatially resolved ensemble of engram cells. Moreover, we revealed a population of peri-engram astrocytes that also exhibit persistent gene-expression changes suggesting that they are ‘engram astrocytes’, and showed that at least one of the astrocyte engram genes, *Igfbp2*, is essential for long-term memory formation. These data help dissect the network of engram cells that consolidate short-term memory to long-term memory and characterize the persistent gene-expression programme that mediates this consolidation.

## Methods

### Mice

All animal experiments were conducted following protocols approved by the Administrative Panel on Laboratory Animal Care at Stanford University. The TRAP2:Ai14 mouse line was a gift from the Luo laboratory at Stanford. TRAP2^10^ mice were heterozygous for the *Fos*^*2A-icreER*^ allele, and homozygous for Ai14 in the C57BL/6 background. *Gt(ROSA)26Sor*^tm1.1(CAG-cas9*,-EGFP)Fezh/J^ mice were acquired from Jackson Laboratory. Mice were group-housed (maximum 5 mice per cage) on a 12 h light:dark cycle (07:00 to 19:00, light) with food and water freely available. Mice were kept with ambient temperature at 21.1 ± 1.1 °C and humidity at 55 ± 5%. Male mice 49–56 days of age were used for all the experiments. Mice were handled daily for 3 days before their first behavioural experiment. The animal protocol no. 20787 was approved by Stanford University APLAC and IACUC. All surgeries were performed under avertin anaesthesia and carprofen analgesia, and every effort was made to minimize suffering, pain and distress.

### Genotyping

The following primers: TCCTGGGCATTGCCTACAAC (forward), CTTCACTCTGATTCTGGCAATTTCG (reverse) and ACCCTGCTGCG CATTG (reporter) were used for genotyping of the *Fos*^*2A-icreER*^ allele; CTGAGCTCACCCACGCT (forward), GGCTGCCTTGCCTTCTCT (reverse), ACTGCTCACAGGGCCAG (reporter) for wild-type allele; CGGCATGGACGAGCTGTA (forward), CAGGGCCGGCCTTGTA (reverse) and AATTGTGTTGCACTTAACG (reporter) were used for genotyping of the Rosa-Ai14 allele; TTCCCTCGTGATCTGCAACTC (forward), CTT TAAGCCTGCCCAGAAGACT (reverse) and CCGCCCATCTTCTAGAAAG (reporter) for Rosa wild-type allele.

### Fear conditioning

The fear conditioning training was conducted according to previously described methods^9^. Each mouse was placed individually in the fear conditioning chamber (Coulbourn Instruments), which was positioned at the centre of a sound-attenuating cubicle. Prior to each session, the chamber was cleaned with 10% ethanol to provide a background odour, while a ventilation fan generated background noise at around 55 dB. The training began with a 2-min exploration period, after which the mice received three tone-foot shock pairings separated by 1-min intervals. Each tone, an 85 dB 2-kHz sound, lasted for 30 s, and was followed by a 2-s foot shock of 0.75 mA, with both ending simultaneously. Following each pairing, the mice remained in the chamber for an additional 60 s before being returned to their home cages. For context recall, the mice were reintroduced to the original conditioning chamber for 5 min, 16 days after the training. Injections of 4-hydroxytamoxifen injections were administered immediately prior to the recall experiments, within 30 min. In the HC and NR groups, 4-hydroxytamoxifen was injected at a similar time to the other two groups during the recall. The behaviour of the mice was recorded and analysed using FreezeFrame software (version 4; Coulbourn Instruments), with motionless bouts lasting over 1 s being considered as freezing.

### Brain tissue dissociation and flow cytometry

Basolateral amygdala was microdissected from live sections cut by a vibratome (300 μm thick). Tissue pieces were enzymatically dissociated using a papain-based digestion system (LK003150, Worthington). In brief, tissue chunks were incubated with papain (containing L-cysteine), DNase I, and kynurenic acid for 1 h at 37 °C and 5% CO_2_. After incubation, tissues were triturated with 300 μm glass pipette tips, then 200 μm glass pipette tips, and 100 μm glass pipette tips. Cell suspensions were then centrifuged at 350*g* for 10 min at room temperature, resuspended in 1 ml EBSS with 10% v/v ovomucoid inhibitor, 4.5% v/v Dnase I, and 0.1% v/v kynurenic acid, and centrifuged again. The supernatant was removed, and 1 ml artificial cerebrospinal fluid (ACSF) was added to the cells. ACSF contained 2.5 mM KCl, 7 mM MgCl2, 0.5 mM CaCl_2_, 1.3 mM NaH_2_PO_4_, 110 mM choline chloride, 24 mM NaHCO_3_, 1.3 mM sodium ascorbate, 20 mM glucose, and 3 mM sodium pyruvate, 2 mM thiourea, and 13.2 mM trehalose. Cells were then passed through a 70 μm cell strainer to remove debris. Hoechst stain (1:2,000; H3570, Life Technologies) was added and incubated in the dark at 4 °C for 10 min. Samples were centrifuged (350*g* for 8 min at 4 °C) and resuspended in 0.5 ml of ACSF and kept on ice for flow cytometry. Live cells were sorted using the BD Vulcan into 384-well plates (Bio-Rad) directly into lysis buffer, oligodT, and layered with mineral oil. After sorting, the plates were immediately snap frozen until reverse transcription.

### Sequencing

The Smartseq3 protocol was used for whole-cell lysis, first-strand synthesis and cDNA synthesis, as previously described with modifications. Following cDNA amplification (23 cycles), the concentration of cDNA was determined via Pico Green quantitation assay (384-well format) and normalized to 0.4 ng μl^−1^ using the TPP Labtech Mosquito HTS and Mantis (Formulatrix) robotic platforms. In-house Tn5 were used for cDNA tagmentation. Libraries were amplified using Kapa HiFi. The libraries were then sequenced on a Novaseq (illumina), using 2 × 100-bp paired-end reads and 2 × 12-bp index reads, with an average of 2 million reads per cell.

### Bioinformatics and data analysis for scRNA-seq

Sequences from Nextseq or Novaseq were demultiplexed using bcl2fastq, and reads were aligned to the mm10 genome augmented with ERCC (External RNA Controls Consortium) sequences, using STARsolo 2.7.9a. We applied standard algorithms for cell filtration, feature selection and dimensionality reduction. In brief, genes that appeared in fewer than five cells, samples with fewer than 2,000 genes and samples with less than 50,000 reads were excluded from the analysis. Out of these cells, those with more than 10% of reads as ERCC or more than 20% mitochondrial were also excluded from analysis. Counts were log-normalized and then scaled where appropriate. Canonical correlation analysis (CCA) function from the Seurat^70^ package was used to align raw data from multiple experiments. The top 20 canonical components were used. After alignment, relevant features were selected by filtering expressed genes to a set of 2,000 with the highest positive and negative pairwise correlations. Genes were then projected into principal component space using the robust principal component analysis. DEG analysis was done by applying the Mann–Whitney–Wilcoxon test on various cell populations.

To find memory-induced genes in each type of neurons, series of strict criteria were applied. First, we removed the background activation by excluding the DEGs resulted from FR versus NF among tdT negative neurons. This guarantees their specificity that DEGs are activity-dependent, rather than a general increase in all cells caused by experience. Second, DEGs must be differentially expressed when FR TRAPed cells are compared to NR and HC controls, ensuring that the DEGs were unique to neuronal ensembles associated with memory recall, and not a result of baseline activity (HC) or activity remaining from the initial fear learning (NR). Finally, each DEG had to meet the criteria of being expressed in a quater of cells and exhibiting at least a 1.75-fold change. By adhering to these standards, a total of 107 DEGs were recognized as ‘remote-memory-associated DEGs’ across 6 distinct neuron types, BLA.Int.Pvalb was not included in the analysis due to insufficient numbers of cells. EnrichR was used for GO, KEGG and REACTOME pathway analysis and classification of enriched genes (log_2_FC > 0.5 and *P* < 0.05) in each subpopulation.

scRNA-seq data from mPFC cells were mapped to mm10 genome with full-length tdTomato construct (including Woodchuck Hepatitis Virus Posttranscriptional sequence included in Ai14 line^71^), which improved the sensitivity in calling tdT^+^ cells. Data from BLA and mPFC cells were integrated using CCA. TRAPed neurons from the each integrated population were analysed, except B-P.Int.Pvalb and B-P.Int.Gpr88 neurons, due to limited cell number. DEGs with *P* < 0.05 (Mann–Whitney–Wilcoxon test) were considered as significant DEGs (highlighted in orange in Fig. 5d and Extended Data Fig. 12f).

After unbiased clustering astrocytes, RNA velocyto^40^ and Monocle3^41^ were applied to infer astrocytic trajectory. DEGs between FR and NF conditions were estimated using Mann–Whitney–Wilcoxon test on each astrocyte population. R, RStudio, Python were used for data analysis.

### RNAscope

The RNAscope multiplex fluorescent reagent kit v2 (323100, ACD) and RNAscope 4-Plex probes were used to conduct the RNAscope experiment according to the manufacturer’s guidelines. The probes employed were either obtained from available stocks or specially created by ACD.

### Gene selection for MERFISH measurements

We used a combination of single-cell RNA sequencing data and literature to select genes for MERFISH. Our selection criteria involved identifying cell-type-marker genes for a particular cell population using a one-vs-all approach. To do this, we performed a Mann–Whitney–Wilcoxon test for each gene between the cells within the cell population and all other cells not in that population, and corrected the resulting *P* values for multiple hypothesis testing to obtain false discovery rate-adjusted *P* values. A gene was considered a cell-type marker for a specific cell population if it met the following criteria: (1) it was expressed in at least 30% of cells within the specified population; (2) the false discovery rate-adjusted *P* value < 0.001; (3) gene expression in the specified population was at least fourfold higher than the average expression in all cells not in that population; and (4) expressed in a fraction of cells within the specified population that was at least 2 times higher than any other population of cells. We then sorted the marker genes for each population by fold change in expression relative to cells outside the population, and saved the top five marker genes for each population to use for marker selection. In addition to these markers, known genes related to microglia, astrocytes and OPCs from the literature and included. Finally, DEGs from remote memory-associated genes were added to the panel with a total number of 158 genes.

### Tissue processing for MERFISH and RNAscope

Brain tissue samples were processed using a fixed-frozen protocol for both MERFISH and RNAscope. In brief, mice were euthanized using CO_2_ and perfused with cold 4% paraformaldehyde. Brain tissue was dissected and followed by incubation at 4 °C in 4% paraformaldehyde overnight, 15% sucrose for 12 h, and 30% sucrose until sink. Brain tissue was frozen in OCT using dry ice and stored at −80 °C until sectioning. Sectioning was performed on a cryostat at −18 °C. Slices were removed and discarded until BLA region was reached.

Slices with 10 μm in thickness were captured onto Superfrost slides for RNAscope and MERSCOPE slides for MERFISH. The same anatomical region was identified for imaging post hoc after sample preparation, before the start of RNAscope or MERFISH imaging.

### Sample preparation and MERFISH imaging

Slides with tissue sections were processed according to MERSCOPE protocol (Vizgen). In brief, slides with tissue sections were washed three times in PBS, and then stored in 70% ethanol at 4 °C for 18 h to permeabilize the tissue. Tissue slices from the same mouse were cut at the same time and distributed onto four coverslips. After permeabilization, the samples were removed from 70% ethanol and washed with Sample Prep Wash Buffer (PN 20300001), then incubated with Formamide Wash Buffer (PN 20300002) at 37 °C for 30 min. Gene Panel Mix (RNA probes) was incubated for 48 h at 37 °C. After hybridization, the samples were washed in Formamide Wash Buffer for 30 min at 47 °C for a total of 2 times to remove excess encoding probes and polyA-anchor probes. Tissue samples were then cleared to remove lipids and proteins that contribute fluorescence background. In brief, the samples were embedded in a thin 4% polyacrylamide gel and were then treated with Clearing Premix (PN 20300003) for 36 h at 37 °C. After digestion, the coverslips were washed in Sample Prep Wash Buffer 2 times and stain with DAPI/PolyT mix for 15 min. Slides were washed with Formamide Wash Buffer followed by Sample Prep Wash Buffer before imaging. Finally, slides were loaded to MERSCOPE Flow Chamber and imaged at both 20× and 63× magnification.

### MERFISH data processing

MERFISH imaging data were processed with MERlin^72^ pipeline with cell segmentation using CellPose^73^, a deep learning-based cell segmentation algorithm based on DAPI staining. Decoding molecules were then assigned to the segmented nuclei to produce a cell-by-gene matrix that list the number of molecules decoded for each gene in each cell. The MERFISH expression matrix for each sample was concatenated with the normalized, log-transformed with Scanpy^74^ and integrated using Harmony^75^ and Leiden^76^ clustering was applied to separate the cells into distinct clusters. TRAPed neurons were assigned based on tdTomato expression. DEGs from a comparison of FR-TRAPed and NF-TRAPed conditions were estimated using Mann–Whitney–Wilcoxon test. Peri-engram cells were computed as follows: for each engram cell (tdT^+^), its peri-engram cells were counted within a radius of 30 μm.

### CalEx injection and behavioural experiments

AAVs carrying CalEx^51^ or tdTomato were generated by Addgene based on the vector pZac2.1-GfaABC_1_D-mCherry-hPMCA2w/b (AAV5, Addgene 111568) or pZac2.1 gfaABC_1_D-tdTomato (AAV5, Addgene 44332). Stereotaxic procedure of viral microinjection has been described previously. In brief, mice with fear training (within 12 h or after 24 h) were anaesthetized and placed onto a stereotaxic frame (model 1900, KOPF). Mice were injected with Carprofen (5 mg kg^−1^) subcutaneously before and after surgery. AAVs carrying hPMCA2w/b (CalEx) or control (tdTomato) viruses were loaded via a glass pipette connected with a 10 μl Hamilton syringe (Hamilton, 80308) on a syringe injection pump (WPI, SP101I) Bevelled glass pipettes (1B100–4; World Precision Instruments) filled with viruses were placed into the BLA (1.3 mm posterior to the bregma, 3.4 mm lateral and to the midline, and 4.6 mm from the pial surface). Either 0.3 μl of AAV5 GfaABC_1_D mCherry-hPMCA2w/b (7 × 10^12^ viral genomes (vg) per ml) or 0.3 μl AAV5 GfaABC_1_D tdTomato (7 × 10^12^ vg ml^−1^) were injected at 100 nl min^−1^. Glass pipettes were withdrawn after 10 min and scalps were cleaned and sutured with sterile surgical sutures. Mice were allowed to recover in clean cages for 7 days. behavioural experiments (recall) were performed three weeks after surgeries. Schematic illustrations (Figs. 1a and 4a,f and Extended Data Fig. 7h,o) created with BioRender.com.

### Open field

Mice were placed in the centre of 40 × 40 cm white box and allowed to freely explore for 15 min. Videos were recorded and analysed by BIOBSERVE III software. The 20 × 20 cm region in the centre was defined as the central zone. The total distance travelled and the activity exploring the centre area were analysed to evaluate the subject’s locomotor ability and anxiety levels.

### Oligos and antibodies

For quantitative PCR analysis, specific primers were designed to amplify the *Igfbp2* gene: *Igfbp2* FW (GTCTACATCCCGCGCTG) and *Igfbp2* RV (GTCTCTTTTCACAGGTACCCG). Additionally, for CRISPR–Cas9 gene editing, six gRNAs (*Igfbp2* guides 1–6) were selected to target distinct regions of the *Igfbp2* gene. These gRNAs were designed based on predicted specificity and efficiency: *Igfbp2* guide 1 (CTACGCT GCTATCCCAACCC), *Igfbp2* guide 2 (GCCAGACGCTCGGGCGT GCA), *Igfbp2* guide 3 (AGAAGGTCAATGAACAGCAC), *Igfbp2* guide 4 (GCCCTCCTGCCGTGCGCACA), *Igfbp2* guide 5 (CTCTCGCACCAGCTCG GCGC), and *Igfbp2* guide 6 (CGTAGCGTCTGGGCGCAGCG).

Antibodies targeting mCherry (Thermo Fisher M11217) and cFOS (Synaptic Systems 226308) were applied for immunostaining following manufacturers’ manuals.

### Inclusion and ethics statement

We, the authors of this manuscript, recognize the importance of inclusion and ethical considerations in scientific research. Our work is guided by the principles of fairness, transparency, and respect for human dignity.

We affirm our commitment to promoting diversity and inclusivity in science, recognizing that diverse perspectives, backgrounds, and experiences enrich research and enhance scientific discovery. We have made efforts to ensure that our study is conducted in a manner that respects and includes individuals of all races, ethnicities, genders, sexual orientations, abilities, and other aspects of human diversity.

We have obtained all necessary ethical approvals and have followed appropriate guidelines and regulations for the research conducted. We have taken measures to protect the privacy and confidentiality of research participants, including obtaining informed consent and ensuring data security.

We acknowledge the potential for harm in scientific research and have taken steps to minimize any potential harm to research participants or others affected by our work. We have carefully considered the potential implications of our research and have taken responsibility for ensuring that our work is conducted in a manner that upholds ethical and moral standards.

We recognize that scientific research has the potential to impact society in profound ways and we are committed to engaging in responsible research practices that promote the well-being of individuals and society as a whole.

In summary, we affirm our commitment to inclusive and ethical research practices and recognize our responsibility to conduct research that is conducted with integrity, respect, and social responsibility.

## Extended Data

**Extended Data Fig. 11 |:**
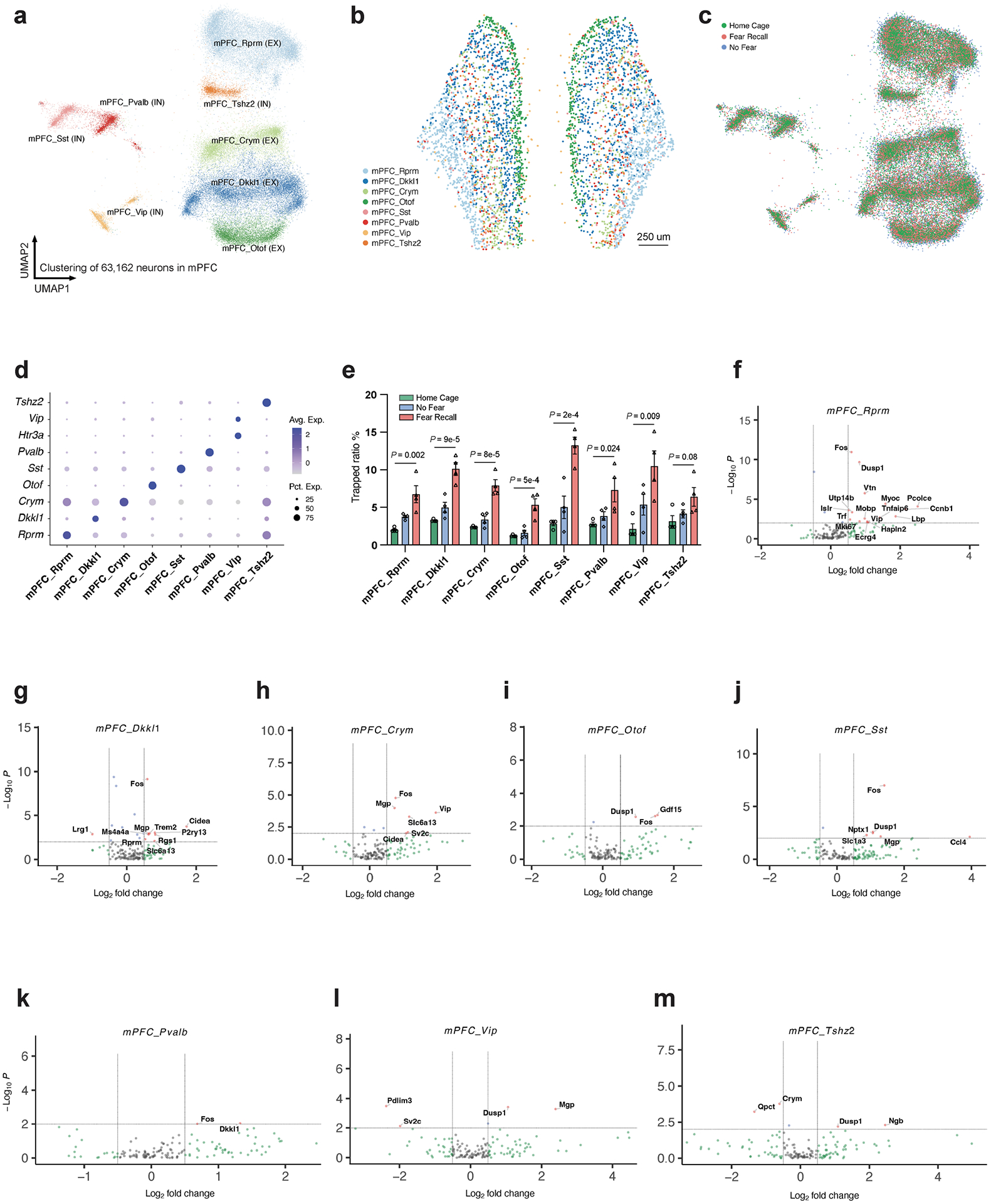
Spatial transcriptomics resolves the memory associated genes in neurons of mPFC. **a)** Clustering of neurons in mPFC from MERFISH data. **b)** Spatial embedding of mPFC neurons. **c)** mPFC neurons grouped by training conditions. **d)** Marker genes of mPFC neurons. **e)** Quantification of tdTomato+ neurons in mPFC, n = 4 mice, mean +/− S.E.M, unpaired two-tailed student t-test. **f**-**m)** DEGs of FR vs NF in TRAPed Rprm neurons (**f**), Dkkl1 neurons (**g**), Crym neurons (**h**), Otof neurons (**i**), Sst neurons (**j**), Pvalb neurons (**k**), Vip neurons (**l**), and Tshz2 neurons (**m**), unadjusted *P* value by Mann Whitney Wilcoxon test. All MERFISH data.

**Extended Data Fig. 12 |:**
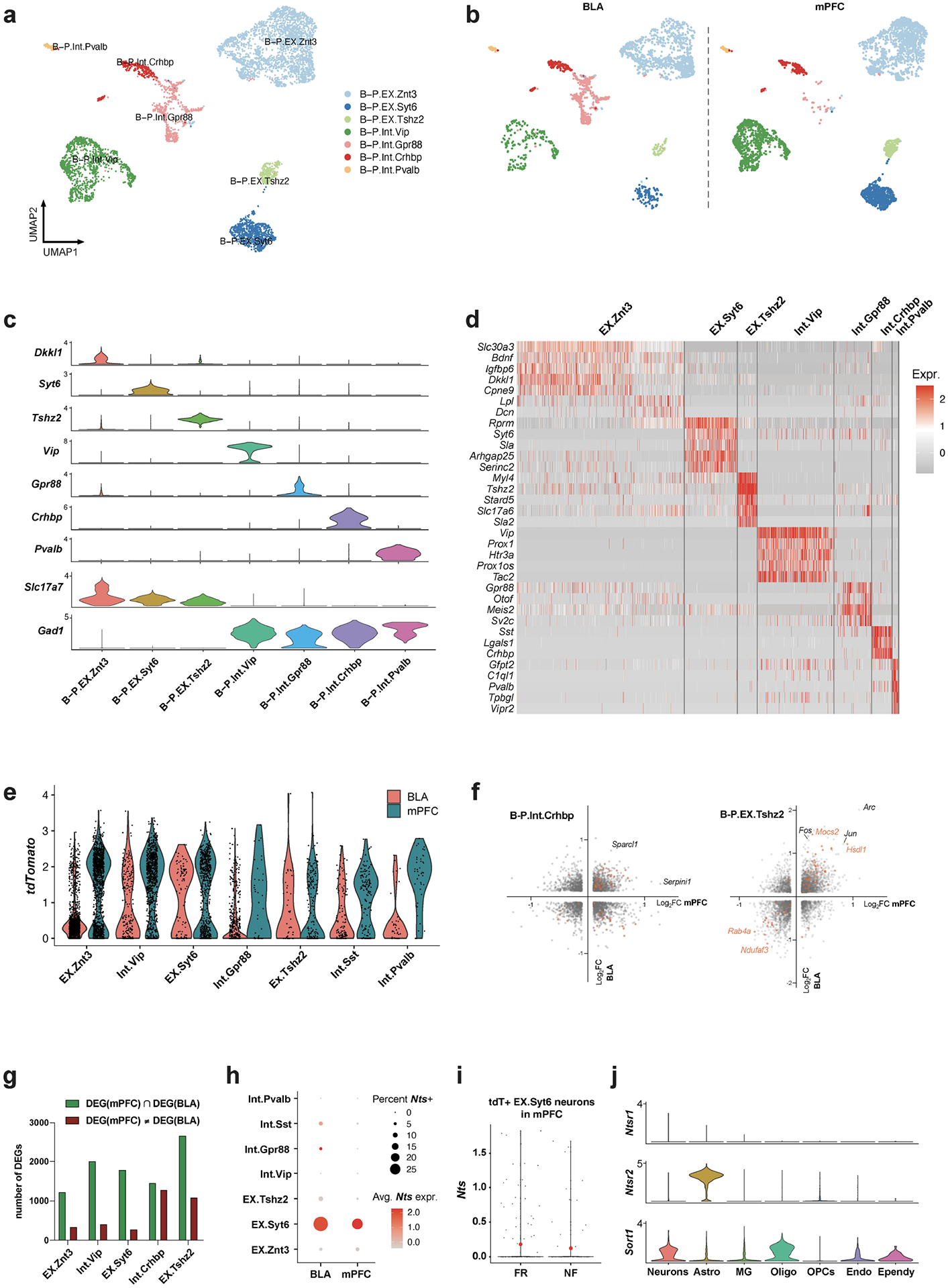
Single- cell transcriptomics resolves the memory associated genes in mPFC and BLA neurons. **a)** Integrated clustering of BLA and mPFC neurons. **b)** Integrated clustering of BLA and mPFC neurons separated by regions. **c)** Distinct markers and *Slc17a7* and *Gad1* expression for each cluster of integrated BLA and mPFC clusters. **d)** Heatmap of top marker genes of integrated BLA and mPFC clusters. **e)** tdTomato expression of integrated BLA and mPFC clusters. **f)** DEGs (FR over NF, TRAPed cells) from BLA and mPFC among B-P.Int.Crhbp and B-P.EX.Tshz2 neurons. **g)** Quantification of DEG numbers in each neuron clusters. **h)** *Nts* expression in each neuron clusters in BLA and mPFC **i)** *Nts* expression in tdT+ B-P.EX.Syt6 neurons from mPFC. **j)** Expression of all three known neurotensin receptors in different cell types of BLA. All scRNAseq data.

**Extended Data Fig. 13 |:**
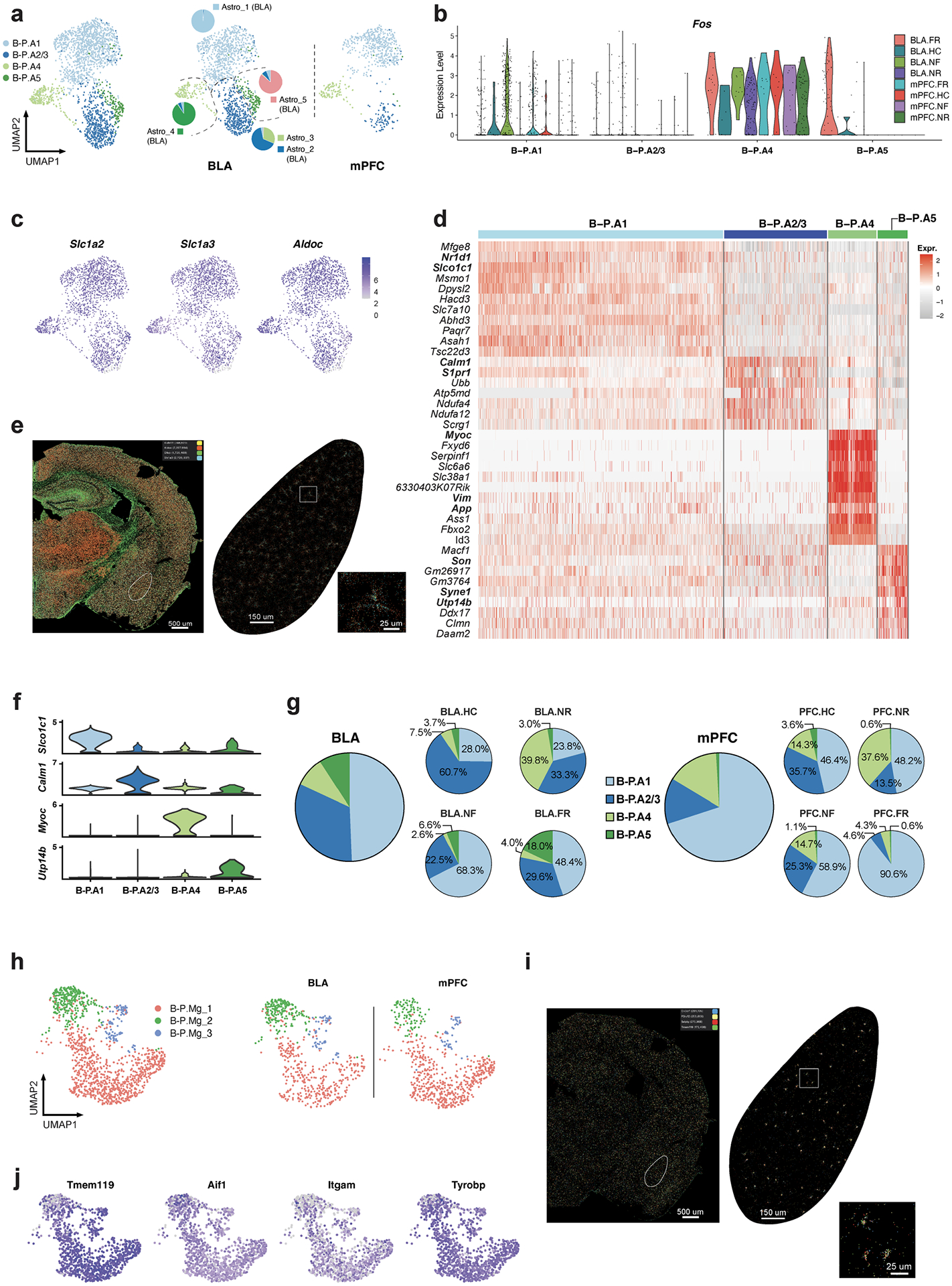
Single-cell transcriptomics resolves the memory associated genes in mPFC and BLA astrocytes and microglia cells. a)Integrated clustering of astrocytes from BLA and mPFC, single-cell RNAseq data. Pie graphs in BLA show the ratio of BLA astrocyte cluster (Astro_1 – 5, Extended Data Fig. 6b). **b)** *Fos* expression separated by astrocyte clusters and condition from BLA and mPFC. **c)** Expression level of astrocyte markers (*Slc1a2*, *Aldoc*, and *Slc1a3*) from BLA and mPFC. **d)** Heatmap of top marker genes of integrated astrocytes cell types from BLA and mPFC. **e)** *Slc1a3*, *Aldh1l1*, *Gfap* and *Aldoc* in situ data from MERFISH. **f)** Distinct markers expression for each cluster of integrated BLA and mPFC astrocyte clusters. **g)** Astrocytes compositions in integrated analysis of mPFC and BLA, separated by conditions. **h)** Integrated clustering of microglia from BLA and mPFC, separated by regions. **i)** *Cx3cr1*, *P2ry12*, *Selplg* and *Tmem119* in situ data from MERFISH. **j)** Expression level of pan microglia markers *Tmem119*, *Aif1*, *Itgam*, and *Tyrobp* from integrated BLA and mPFC. All scRNAseq data, except **e** and **j**.

## Supplementary Material

Supplementary Information

## Data Availability

The scRNA-seq data are available at GSE246147 and MERFISH data are available at https://doi.org/10.6084/m9.figshare.24424801. Materials are available upon reasonable request. Source data are provided with this paper.
