# Supplementary material for: Spatial transcriptomics reveal neuron–astrocyte synergy in long-term memory
Source: Nature. Author manuscript; Available in PMC 2024 Mar 14. (PMC10937396; doi:10.1038/s41586-023-07011-6)
Supplement: Supplementary Information [file NIHMS1971415-supplement-Supplementary_Information.pdf]

nature portfolio

https://doi.org/10.1038/s41586-023-07011-6

Supplementary information

Spatial transcriptomics reveal neuron–astrocyte synergy in long-term memory

In the format provided by the authors and unedited

Supplemental notes

Beyond the aforementioned pathways, many DEGs are also immediate early genes (IEGs), indicating that fear memory selectively modulates activation. We next explored the differentiation between cells that initiate IEG activity and those that remain inactive. First, we employed *Fos* as an indicator of activation and discovered that the expression of *Dusp1*, *Npas4*, and *Egr1* bore the strongest correlation with *Fos* expression across all neurons, or within TRAPed population in the basolateral amygdala (Supplemental Fig. 2**a**-**d**). These four genes are recognized IEGs in the literature. We then allocated an 'IEG score' to neurons based on the expression of these genes and pinpointed those genes displaying differential expression between IEG active cells and IEG negative cells (Supplemental Fig. 2**e**-**j**, Supplemental Fig. 3**a**-**i**). In addition to that, we examined neurons activated for the first-time during recall versus neurons undergoing reactivation. Notably, among the active neurons (IEG_high), the FR condition exhibited higher tdTomato expression compared to the remaining three conditions, implying the existence of reactive neurons within the FR condition (Supplemental Fig. 4**a**-**c**). Subsequent differential gene expression analysis between first-time active neurons (IEG_high; tdTomato low) and reactivated neurons (IEG_high; tdTomato high) revealed a considerable number of DEGs in the FR condition, but not in the NF condition (Supplemental Fig. 4**d**-**j**).

Supplementary Figure 5 shows our FACS strategy for sorting cells from the brain.

SI Table lists the top 20 genes of interest in this paper.

Supplemental Figure 1

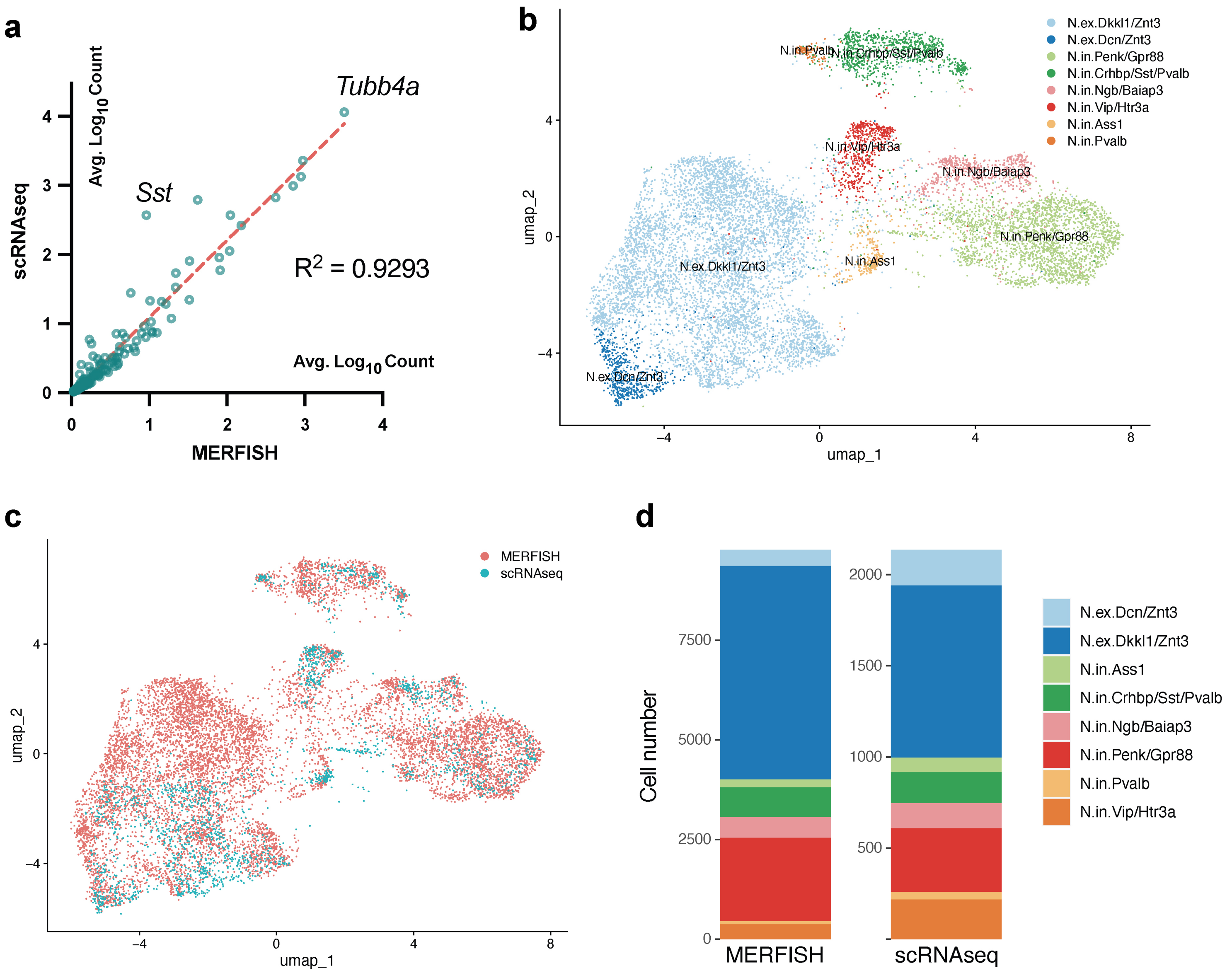

Supplemental Figure 1

- **a)** Correlation analysis of gene expression measured by MERFISH and scRNAseq. R squared is calculated using simple linear regression model.
- **b)** Integrated clustering of all neurons from MERFISH and scRNAseq.
- **c)** Integrated clustering of all neurons from MERFISH and scRNAseq, colored by methods.
- **d)** Neuronal cluster identified by MERFISH and scRNAseq.

Supplemental Figure 2

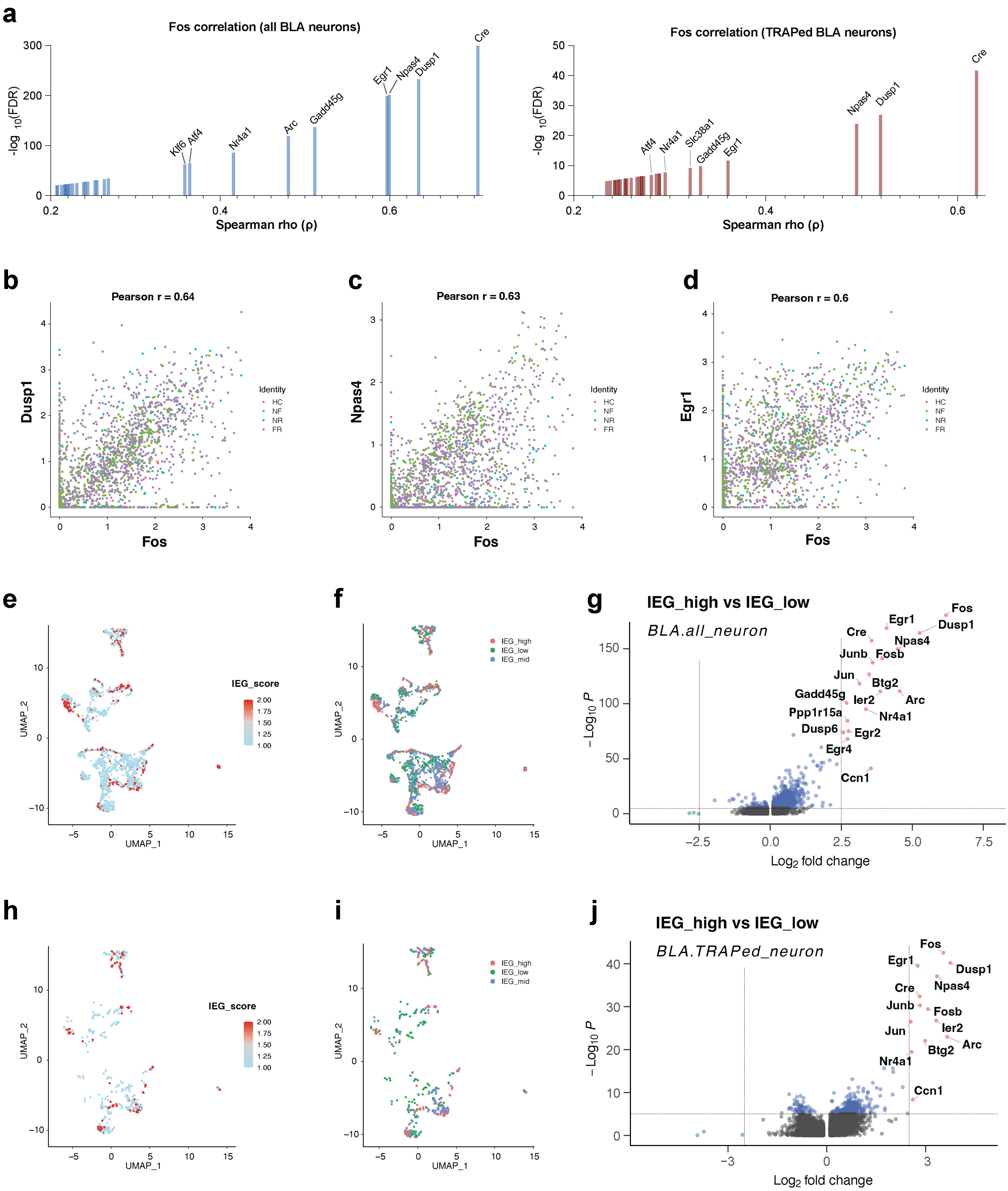

Supplemental Figure 2

- **a)** Genes with high correlation *Fos* expression of all neurons (left) or TRAPed neurons (right). Correlations were assessed by Spearman's rank correlation coefficient.
- **b)** Pearson's correlation of *Dusp1* and *Fos* in all neurons
- **c)** Pearson's correlation of *Npas4* and *Fos* in all neurons
- **d)** Pearson's correlation of *Egr1* and *Fos* in all neurons
- **e)** Feature plot of IEG_score (expression of *Fos*, *Dusp1*, *Npas4*, and *Egr1*) in all neurons.
- **f)** All neurons grouped by IEG_high (top quarter), IEG_low (bottom quarter) and IEG_mid (25% - 75%).
- **g)** DEG analysis of all neurons in BLA, IEG_high over IEG_low, unadjusted *P* value by Mann Whitney Wilcoxon test.
- **h)** Feature plot of IEG_score in TRAPed neurons.
- **i)** TRAPed neurons grouped by IEG_high, IEG_low, and IEG_mid.
- **j)** DEG analysis of TRAPed neurons in BLA, IEG_high over IEG_low, unadjusted *P* value by Mann Whitney Wilcoxon test.

  All scRNAseq data.

Supplemental Figure 3

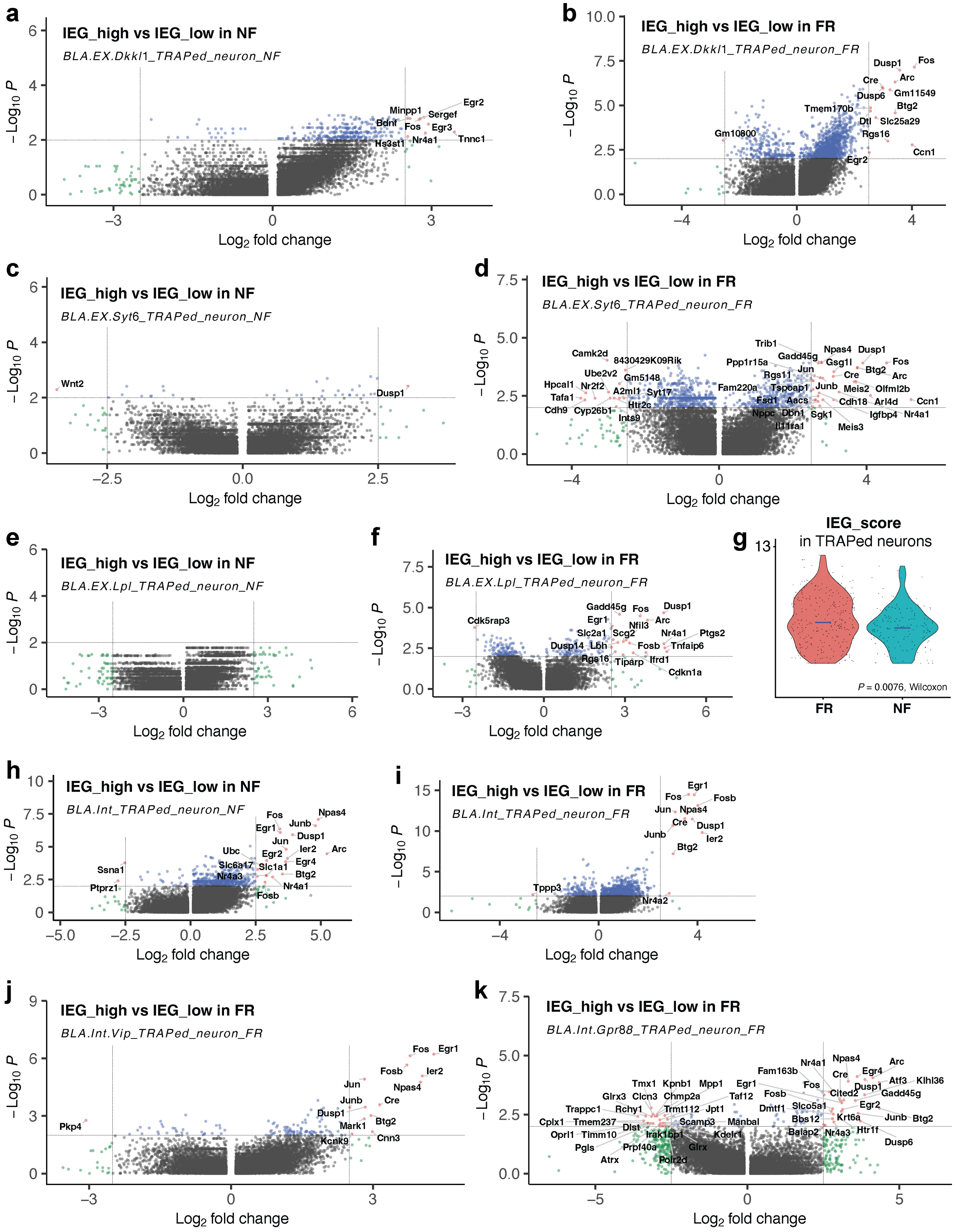

Supplemental Figure 3

- **a)** Differential gene expression analysis of IEG_high over IEG_low cells in NF condition, among BLA.EX.Dkkl1 TRAPed neurons.
- **b)** Differential gene expression analysis of IEG_high over IEG_low cells in FR condition, among BLA.EX.Dkkl1 TRAPed neurons.
- **c)** Differential gene expression analysis of IEG_high over IEG_low cells in NF condition, among BLA.EX.Syt6 TRAPed neurons.
- **d)** Differential gene expression analysis of IEG_high over IEG_low cells in FR condition, among BLA.EX. Syt6 TRAPed neurons.
- **e)** Differential gene expression analysis of IEG_high over IEG_low cells in NF condition, among BLA.EX.Lpl TRAPed neurons.
- **f)** Differential gene expression analysis of IEG_high over IEG_low cells in FR condition, among BLA.EX.Lpl TRAPed neurons.
- **g)** IEG_score plot of TRAPed neuron in the BLA, two-sided Mann Whitney Wilcoxon test.
- **h)** Differential gene expression analysis of IEG_high over IEG_low cells in NF condition, among BLA inhibitory TRAPed neurons.
- **i)** Differential gene expression analysis of IEG_high over IEG_low cells in FR condition, among BLA. inhibitory TRAPed neurons.
- **j)** Differential gene expression analysis of IEG_high over IEG_low cells in FR condition, among BLA.Int.Vip TRAPed neurons.
- **k)** Differential gene expression analysis of IEG_high over IEG_low cells in FR condition, among BLA.Int.Gpr88 TRAPed neurons.

All scRNAseq data, unadjusted *P* value by Mann Whitney Wilcoxon test.

Supplemental Figure 4

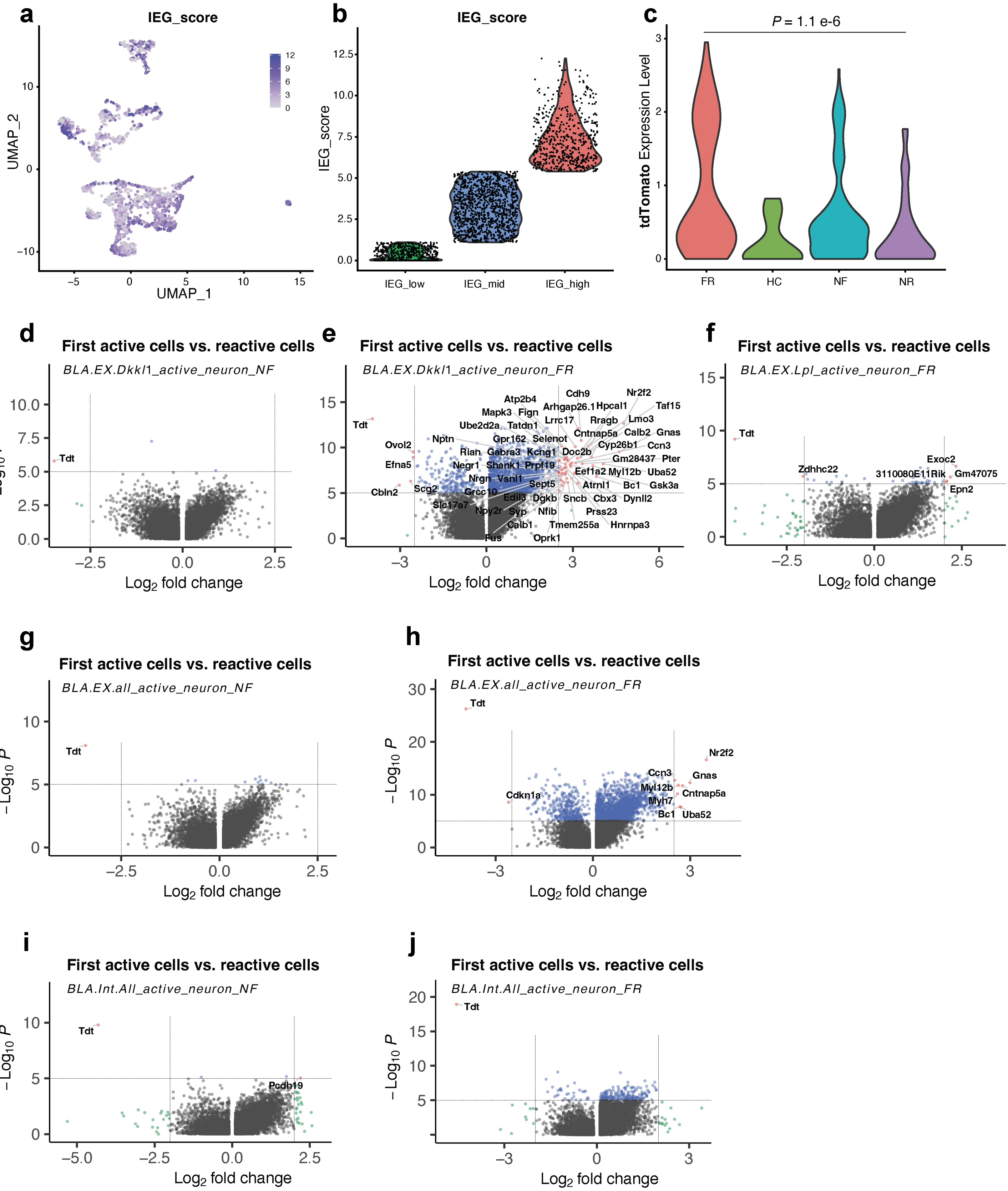

Supplemental Figure 4

a) IEG score of all neurons in BLA.
b) IEG score of all neurons in BLA grouped by IEG_high, IEG_low, and IEG_mid.
c) tdTomato expression of four training conditions, among IEG_high neurons in BLA, one-way ANOVA, $F = 10.25$.
d) Differential gene expression analysis of first active cells over reactive cells in NF condition, among BLA.EX.Dkkl1 IEG_high neurons.
e) Differential gene expression analysis of first active cells over reactive cells in FR condition, among BLA.EX.Dkkl1 IEG_high neurons.
f) Differential gene expression analysis of first active cells over reactive cells in FR condition, among BLA.EX.Lpl IEG_high neurons.
g) Differential gene expression analysis of first active cells over reactive cells in NF condition, among BLA excitatory IEG_high neurons.
h) Differential gene expression analysis of first active cells over reactive cells in FR condition, among BLA excitatory IEG_high neurons.
i) Differential gene expression analysis of first active cells over reactive cells in NF condition, among BLA inhibitory IEG_high neurons.
j) Differential gene expression analysis of first active cells over reactive cells in FR condition, among BLA inhibitory IEG_high neurons.
All scRNAseq data, unadjusted *P* value by Mann Whitney Wilcoxon test.

Supplemental Figure 5

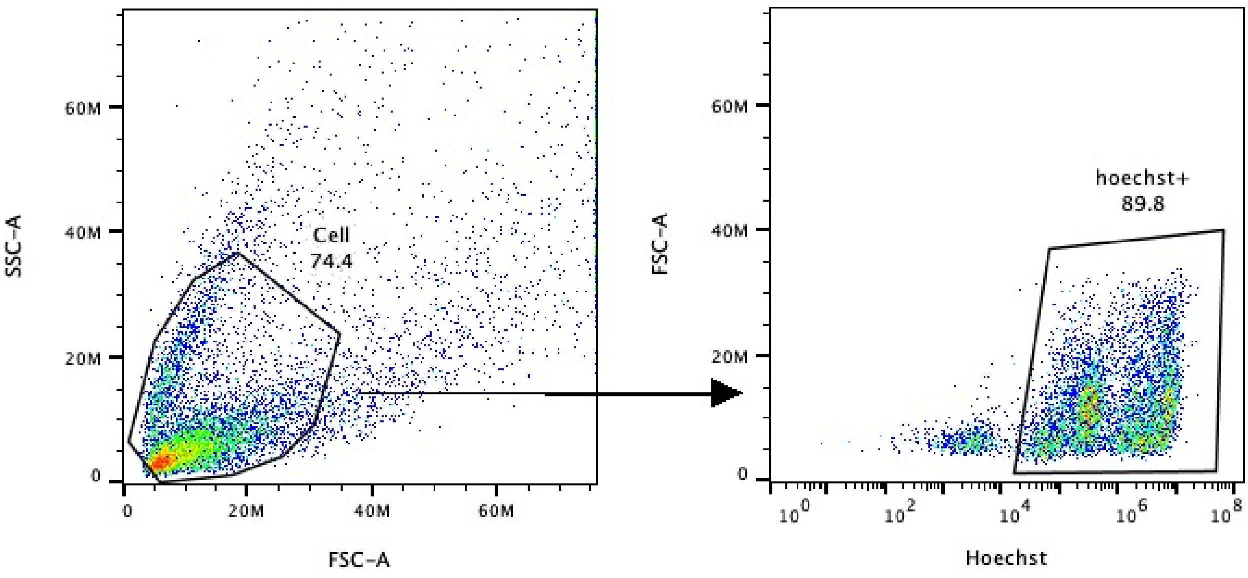

Supplemental Figure 5 FACS strategy
I first identify the population of events that contain cells in the forward vs. side scatter area plot. Next, I selected hoechst+ cells based on hoechst intensity and the cell morphology. Finally, I collected the cells in 384-well plates.

SI Table

Penk	Penk (encoding proenkephalin) was induced in both excitatory and inhibitory engram neurons in the BLA following fear-memory recall.	Proenkephalin is processed to yield enkephalin, an endogenous opioid. This binds and activates opioid receptors in the brain and is implicated in fear memory consolidation, extinction, anxiety-like behaviors, and stress response.
Sv2c	Sv2c (encoding synaptic vesicle glycoprotein 2C) showed elevated expression in the FR condition compared to the NF condition among inhibitory engram neurons in the BLA.	Synaptic vesicle glycoprotein 2C is recognized for its role in neurotransmitter release at synapses, it modulates synaptic function and neurotransmitter release.
Plk2	Plk2 (encoding polo-like kinase 2) was upregulated in engram neurons of both BLA and mPFC.	Polo-like kinase 2 (Plk2) is a transcriptional target of Npas4, Plk2 influences synapse formation, stabilization of long-term synaptic plasticity, and contextual fear memory.
Trim32	Trim32 (encoding tripartite motif-containing protein 32) was upregulated in engram neurons of both BLA and mPFC.	Tripartite motif-containing protein 32 is an E3 ubiquitin ligase, has vital for protein degradation, synapse formation, and remodeling. Its Drosophila ortholog, Thin, modulates homeostatic plasticity via neurotransmitter release repression.
Ubl3	Ubl3 (encoding Ubiquitin Like 3) was upregulated in engram neurons of both BLA and mPFC.	Ubl3 is a ubiquitin-like protein family member, it's implicated in cellular processes via covalent attachment to target proteins, specifically influencing protein sorting to small extracellular vesicles.
Ubc	Ubc (encoding Ubiquitin) was upregulated in engram neurons of both BLA and mPFC.	Ubiquitin C: Central to protein degradation in the ubiquitin-proteasome system. By marking substrates, it manages the abundance of synaptic receptors, thereby affecting synaptic strength.
Mal2	Mal2 (encoding Myelin and lymphocyte protein 2) was upregulated in engram neurons of both BLA and mPFC.	Mal2 is an integral membrane component of synaptic vesicles linked with vGlut1-positive nerve terminals.
Scg2	Scg2 (encoding secretogranin 2) displayed increased expression in engram Gpr88 neurons in the BLA when comparing the FR to NF conditions.	Secretogranin II (Scg2) is a member of the granin family, which are acidic proteins found in secretory granules of a variety of endocrine and neuroendocrine cells. Scg2 was shown to be involved in the processing and packaging of neuropeptides within secretory granules. Scg2 was recently identified with an instructive role in establishing the network of Fos-activated neurons.
Pcsk1n	Pcsk1n (encoding ProSAAS) was upregulated in the FR condition among engram neurons of both Sst and Vip inhibitory types in the BLA.	ProSAAS-derived peptides and PC1/3 are co-localized in neuroendocrine cells and neurons. PC1/3 is responsible for the maturation of many neuropeptides, which are critical for various neural functions. ProSAAS was reported to inhibit prohormone processing and be required for fear memory.
Npas4	Npas4 (encoding Neuronal PAS domain protein 4) showed elevated expression in the FR engram neurons of Vip and Calm1 inhibitory types in the BLA.	Npas4 is a Ca2+ influx dependent gene that regulates synapse development in inhibitory neurons by regulating activity-dependent genes, marks a subset of fear induced engram neurons in parallel of Fos engrams, is required for both short-term and long-term contextual fear memory.
Egr1	Egr1 (encoding early growth response 1) was upregulated in the FR engram neurons of Vip and Calm1 inhibitory types in BLA.	Egr1 was reported to be required in lateral amygdala for long-term fear memory consolidation without impairing acquisition or short-term memory

Dusp1	Dusp1 (encoding dual specificity phosphatase 1) was upregulated in the engram neurons of Vip inhibitory neurons in BLA.	Dusp1's role in neurons include regulating mitogen-activated protein kinases (MAPKs) signaling pathways, specifically the extracellular signal-regulated kinase (ERK) pathway. The ERK pathway is implicated in synaptic plasticity, which is the cellular foundation of learning and memory.
Ier2	Ier2 (encoding early immediate early response 2) was upregulated in the engram neurons of Vip inhibitory neurons in the BLA.	Ier2 have been implicated in mediating synaptic strength and neuronal development.
Nts	Nts (encoding neurotensin) demonstrated elevated expression in engram neurons in the FR condition compared to the NF condition within the Syt6 neurons in the BLA.	Neurotensin is a neuropeptide that modulate dopamine release and pain signals. It was shown to modulate associative memory in paraventricular thalamus to BLA circuit.
Ntsr2	Ntsr2 (encoding neurotensin receptor 2) was predominantly expressed in astrocytes.	Ntsr2 as a receptor for neurotensin, was shown to be essential for contextual fear memory.
Creb1	The Creb1 pathway (encoding cAMP responsive element binding protein 1) emerged as the most activated by fear memory in engram neurons in the BLA, involving 18 genes such as Klf6, Junb, and Dusp1.	The CREB signaling pathway is widely implicated in long-term memory consolidation.
Tac1	Tac1 (encoding tachykinin precursor 1) showed reduced expression in the engram neurons of the FR condition compared to the NF condition within specific neuron types in the BLA.	One of the derivatives of Tachykinin Precursor 1, Substance P is involved in pain perception.
Tac2	Tac2 (encoding tachykinin 2 also known as neurokinin B) was downregulated in the engram neurons of FR condition as compared to the NF condition, in the inhibitory neurons of BLA.	Neurokinin B has been implicated in various physiological processes, including reproductive function, thermoregulation, pain, and stress. In the BLA, tachykinin receptor 3 (the receptor of neurokinin B) activation leads to neuronal excitation and enhanced fear-potentiated startle
Cartpt	Cartpt (encoding cocaine- and amphetamine-regulated transcript protein) was downregulated in engram neurons of Sst and Calm1 inhibitory neurons in the BLA.	Cartpt is modulated by drugs such as cocaine and amphetamine, it has been found to act both as a neurotransmitter and influence the activity of other neurotransmitters.
Igfbp2	Igfbp2 (encoding insulin-like growth factor binding protein 2) was found enriched in peri-engram astrocytes in both mPFC and BLA.	Igfbp2, an astrocytic secreted protein, was reported to have multiple effects on neurons, including changes in synaptic transmission and excitability. A peptide derived from Igfbp2 was shown to enhance neuroplasticity. Igfbp2 is involved in fear memory formation in the BLA.
