# Supplementary material for: Spatial transcriptomics reveal neuron–astrocyte synergy in long-term memory
Source: Nature. 2024 Feb 7;627(8003):374–81. doi: 10.1038/s41586-023-07011-6 (PMC10937396; doi:10.1038/s41586-023-07011-6)
Supplement: Supplementary file 1 [file 41586_2023_7011_MOESM2_ESM.pdf]

Corresponding author(s): Stephen R. Quake,T homas c. Sudhof

Last updated by author(s): Oct 26, 2023

Reporting Summary

Nature Portfolio wishes to improve the reproducibility of the work that we publish. This form provides structure for consistency and transparency in reporting. For further information on Nature Portfolio policies, see our Editorial Policies and the Editorial Policy Checklist.

Statistics

For all statistical analyses, confirm that the following items are present in the figure legend, table legend, main text, or Methods section.

n/a	Confirmed	
☐	☒	The exact sample size (*n*) for each experimental group/condition, given as a discrete number and unit of measurement
☐	☒	A statement on whether measurements were taken from distinct samples or whether the same sample was measured repeatedly
☐	☒	The statistical test(s) used AND whether they are one- or two-sided<br>*Only common tests should be described solely by name; describe more complex techniques in the Methods section.*
☐	☒	A description of all covariates tested
☐	☒	A description of any assumptions or corrections, such as tests of normality and adjustment for multiple comparisons
☐	☒	A full description of the statistical parameters including central tendency (e.g. means) or other basic estimates (e.g. regression coefficient) AND variation (e.g. standard deviation) or associated estimates of uncertainty (e.g. confidence intervals)
☐	☒	For null hypothesis testing, the test statistic (e.g. *F*, *t*, *r*) with confidence intervals, effect sizes, degrees of freedom and *P* value noted<br>*Give P values as exact values whenever suitable.*
☒	☐	For Bayesian analysis, information on the choice of priors and Markov chain Monte Carlo settings
☒	☐	For hierarchical and complex designs, identification of the appropriate level for tests and full reporting of outcomes
☐	☒	Estimates of effect sizes (e.g. Cohen's *d*, Pearson's *r*), indicating how they were calculated

Our web collection on statistics for biologists contains articles on many of the points above.

Software and code

Policy information about availability of computer code

Data collection	Freeze Frame software (version 4; Coulbourn Instruments); BIOBSERVE Ill (Version 3.01); Leica SP8; Olympus VS200 ASW 3.2.1 (Olympus); Novaseq6000 (illumina); Merscope (Vizgen)
Data analysis	Viewer Ill (Biobserve); BIOBSERVE Ill (Version 3.01); Olympus OlyVIA (3.2.1); Fiji (ImageJ2 V2.9.0/l.53t); GraphPad Prism 9; R (version 4.2.2); Rstudio (2022.12.0+353); Seurat (4.3); Python (3.9.10); Scanpy (l.9.1); Monocle 3; velocyto.R (git: velocyto-team/velocyto.R, Dec 6, 2019 version); MERiin (git: ZhuangLab/MERlin, Apr 20, 2020 version); Cellpose 2.0; Harmony (git: immunogenomics/harmony, version 0.1.1); Leiden (git: vtraag/leidenalg, version 0.4.3)

For manuscripts utilizing custom algorithms or software that are central to the research but not yet described in published literature, software must be made available to editors and reviewers. We strongly encourage code deposition in a community repository (e.g. GitHub). See the Nature Portfolio guidelines for submitting code & software for further information.

Data

Policy information about availability of data

All manuscripts must include a data availability statement. This statement should provide the following information, where applicable:

- Accession codes, unique identifiers, or web links for publicly available datasets
- A description of any restrictions on data availability
- For clinical datasets or third party data, please ensure that the statement adheres to our policy

Transcriptomics data are available in 10.6084/m9.figshare.24424801 and GSE246147.

Research involving human participants, their data, or biological material

Policy information about studies with human participants or human data. See also policy information about sex, gender (identity/presentation), and sexual orientation and race, ethnicity and racism.

Reporting on sex and gender	N/A
Reporting on race, ethnicity, or other socially relevant groupings	N/A
Population characteristics	N/A
Recruitment	N/A
Ethics oversight	N/A

Note that full information on the approval of the study protocol must also be provided in the manuscript.

Field-specific reporting

Please select the one below that is the best fit for your research. If you are not sure, read the appropriate sections before making your selection.

☒ Life sciences ☐ Behavioural & social sciences ☐ Ecological, evolutionary & environmental sciences

For a reference copy of the document with all sections, see nature.com/documents/nr-reporting-summary-flat.pdf

Life sciences study design

All studies must disclose on these points even when the disclosure is negative.

Sample size	No statistical methods were used to predetermine sample sizes. Sample sizes (n = 8-10 mice) in behavior tests were based on work in previous publications (pmid 35859170). For imaging experiments, at least three animals per genotype or condition were used. For single-cell RNA sequencing experiment, 4-5 mice per group were sequenced. For merfish sequencing experiment, 4-5 mice per group were imaged. For transcriptomics experiments, sample size were based on work in previous publication (pmid 33177708).
Data exclusions	All data were included
Replication	All replicates reproduced the phenotypes. At least three independent cohorts were performed. The total number of animals and cells is reported for all experiments.
Randomization	Animals were randomized by cage prior to surgeries or behavior trainings.
Blinding	Behavioral experiment were performed blind of AAV injection groups. Transcriptomics experiments and sequencing were performed blind of training conditions.

Reporting for specific materials, systems and methods

We require information from authors about some types of materials, experimental systems and methods used in many studies. Here, indicate whether each material, system or method listed is relevant to your study. If you are not sure if a list item applies to your research, read the appropriate section before selecting a response.

Materials & experimental systems

n/a	Involved in the study	
☐	☒	Antibodies
☒	☐	Eukaryotic cell lines
☒	☐	Palaeontology and archaeology
☐	☒	Animals and other organisms
☒	☐	Clinical data
☒	☐	Dual use research of concern
☒	☐	Plants

Methods

n/a	Involved in the study	
☒	☐	ChIP-seq
☐	☒	Flow cytometry
☒	☐	MRI-based neuroimaging

Antibodies

Antibodies used	mCherry (Thermo Fisher M11217) and cFOS (Synaptic System 226308)
Validation	Antibodies have been validated by manufacturers.

Animals and other research organisms

Policy information about studies involving animals; ARRIVE guidelines recommended for reporting animal research, and Sex and Gender in Research

Laboratory animals	Fos2A-iCreER and Ai14 in the C57BL/6 background. C57BL/6J (Jax stock #: 00064) and Gt(ROSA)26Sortm1.1(CAG-cas9*,-EGFP)Fezh/J (Jax stock #024858) mice were directly purchased from The Jackson Laboratory. Mice were housed in groups with up to five mice per cage and on 12-hour light-dark cycles (7 am to 7 pm, light), before behavior experiments took place. Animals are kept with ambient temperature at 70 +/- 2 F and humidity at 55% +/- 5%. Male mice 49–56 days of age were used for all the experiments.
Wild animals	No wild animals were used in the study.
Reporting on sex	Only male mice were included
Field-collected samples	No field collected samples were used in the study.
Ethics oversight	The animal protocol #20787 was approved by Stanford University APLAC and IACUC

Note that full information on the approval of the study protocol must also be provided in the manuscript.

Plants

Seed stocks	No plants were involved in this study.
Novel plant genotypes	No plants were involved in this study.
Authentication	No plants were involved in this study.

Flow Cytometry

Plots

Confirm that:

☒ The axis labels state the marker and fluorochrome used (e.g. CD4-FITC).

☒ The axis scales are clearly visible. Include numbers along axes only for bottom left plot of group (a 'group' is an analysis of identical markers).

☒ All plots are contour plots with outliers or pseudocolor plots.

☒ A numerical value for number of cells or percentage (with statistics) is provided.

Methodology

Sample preparation	Flow Cytometry was applied to distribute individual cells to 384 plates.<br><br>Basal lateral amygdala was microdissected using a live vibratome sections (300 µm thick). Tissue pieces were enzymatically dissociated using a papain-based digestion system (LK003150, Worthington). Briefly, tissue chunks were incubated with papain (containing L-cysteine), DNase I, and kynurenic acid for 1 hour at 37°C and 5% CO2. After incubation, tissues were triturated with 300 um glass pipette tips, then 200 um glass pipette tips, and 100 um glass pipette tips. Cell suspensions were then centrifuged at 350g for 10 minutes at room temperature, resuspended in 1 ml EBSS with 10% v/v ovomucoid inhibitor, 4.5% v/v Dnase I, and 0.1% v/v kynurenic acid, and centrifuged again. The supernatant was removed, and 1 ml ACSF was added to the cells. ACSF contained 1 mM KCl, 7 mM MgCl2, 0.5 mM CaCl2, 1.3 mM NaH2PO4, 110 mM choline chloride, 24 mM NaHCO3, 1.3 mM Na ascorbate, 20 mM glucose, and 0.6 mM sodium pyruvate. Cells were then passed through a 70-µm cell strainer to remove debris. Hoechst stain (1:2,000; H3570, Life Technologies) was added and incubated in the dark at 4°C for 10 minutes. Samples were centrifuged (350g for 8 minutes at 4°C) and resuspended in 0.5 ml of ACSF and kept on ice for flow cytometry. Live cells were sorted using the BD Vulcan into 384-well plates (Bio-Rad) directly into lysis buffer, oligodT, and layered with mineral oil. After sorting, the plates were immediately snap frozen until reverse transcription.
Instrument	BD Vulcan
Software	BD Vulcan
Cell population abundance	Intact cells were sorted out of debris and dead cells
Gating strategy	I first identify the population of events that contain cells in the forward vs. side scatter area plot. Next, I selected hoechst+ cells based on hoechst intensity and the cell morphology. Finally, I collected the cells in 384-well plates.

☒ Tick this box to confirm that a figure exemplifying the gating strategy is provided in the Supplementary Information.
